# DNMT3a promotes LUAD cell proliferation and metastasis by activating the HDAC7 signalling pathway

**DOI:** 10.7150/ijbs.96509

**Published:** 2025-01-27

**Authors:** Menglong Jiang, Xin Zhou, Yingtong Feng, Peng Ding, Jinfeng Li, Di Lu, Jiapei Qin, Yibing Bai, An Wang, Chunfang Xia, Jinliang Wang, Xiaolong Yan, Zhiqiang Ma, Renquan Zhang

**Affiliations:** 1 Department of Thoracic Surgery, the First Affiliated Hospital of Anhui Medical University, Hefei 230022, China.; 2 Department of Medical Oncology, Senior Department of Oncology, the Fifth Medical Center, Chinese PLA General Hospital, Beijing 100853, China.; 3 The Second Medical Center, Chinese PLA General Hospital, Beijing 100853, China.; 4 Department of Cardiothoracic Surgery, the Affiliated Huaihai Hospital of Xuzhou Medical University/the 71st Group Army Hospital of PLA, Xuzhou 221004, China.; 5 Department of Thoracic Surgery, Tangdu Hospital, the Fourth Military Medical University, Xi'an 710038, China.

**Keywords:** LUAD, DNMT3a, HDAC7, ZEB1, c-Myc

## Abstract

**Background:** Changes in DNA methylation patterns, in which DNA methyltransferases such as DNA methyltransferase 3 alpha (DNMT3a) play important roles, are closely related to the occurrence and development of tumours. However, the role and mechanism of DNMT3a in lung adenocarcinoma (LUAD) remain unknown. The aim of this study was to investigate the potential effect of DNMT3a on LUAD cell proliferation and metastasis and explore the underlying molecular mechanism.

**Methods:** Immunohistochemistry and Kaplan‒Meier survival analysis were used to investigate the relationship between the expression of DNMT3a and histone deacetylase 7 (HDAC7) and the survival, prognosis and clinicopathological features of patients. The effects of DNMT3a on the proliferation and metastasis of LUAD cells were studied *in vivo* and *in vitro*. Recombinant lentivirus-mediated *in vitro* gene overexpression or knockdown, western blotting, Quantitative real-time polymerase chain reaction (qRT‒PCR) and other methods were used in this study to elucidate the potential molecular mechanisms by which DNMT3a promotes LUAD cell proliferation and metastasis.

**Results:** High expression of DNMT3a or HDAC7 was positively correlated with poor prognosis, high AJCC 8th edition stage, and poor tumour differentiation in LUAD patients. LUAD patients with DNMT3a/HDAC7 co-low expression exhibited the worst prognosis. Upregulation of DNMT3a can promote LUAD cell proliferation and metastasis by upregulating HDAC7 and further activating the expression of downstream mediators ZEB1 and c-Myc. Conversely, overexpression of HDAC7 reversed the attenuation of tumour growth and metastasis and the suppression of c-Myc and ZEB1 expression mediated by downregulation of DNMT3a, further indicating the existence of positive feedback regulation between DNMT3a and HDAC7 in LUAD.

**Conclusion:** Our findings first confirmed that DNMT3a acts as a tumour promoter inducing malignant progression of LUAD by upregulating HDAC7 and further inducing upregulation of ZEB1 and c-Myc. Targeting DNMT3a along with HDAC7 might be a promising therapeutic strategy for LUAD.

## 1. Introduction

Tumour development and progression are accompanied by changes in DNA methylation patterns^1^. The biological role of DNA methylation, one of the most extensively characterized epigenetic modifications, is to maintain DNA in a transcriptionally quiescent state, resulting in gene silencing^2^. DNA methylation refers to the chemical modification process in which a methyl (CH_3_) group transferred from the methyl donor S-adenosyl methionine (SAM) binds covalently to a specific base in a DNA sequence^3^. This process is dependent on the action of DNA methyltransferases (DNMTs), enzymes that catalyse the addition of methyl groups to the 5' carbon of cytosine residues^4^. Importantly, compared with normal cells of the same tissue type, tumour cells always exhibit a decreased overall genome-wide methylation level and an increased *de novo* methylation level of select CpG islands^1^. DNMT3a (DNA methyltransferase 3 alpha) is the *de novo* methyltransferase responsible for DNA methylation of CpG dinucleotides^5^, and it was reported to be the methyltransferase most frequently mutated in tumours^6^. DNMT3a mutation has been observed in haematologic malignancies such as acute myeloid leukaemia (AML), in which it is associated with poor prognosis^7^, ^8^. However, in lung adenocarcinoma (LUAD), the relationship between the expression level of DNMT3a and clinical prognosis remains to be explored, and the molecular mechanism by which DNMT3a mediates malignant tumour progression remains unclear.

Importantly, the epigenomic landscape that determines the transcriptional activity of genes includes not only the establishment of DNA methylation but also modifications of chromatin that regulate transcription, especially histone modification^9^. DNA methylation interacts with histone modification, and this interaction is a dynamic process that regulates gene transcription^10^. Methylated cytosines resulting from DNA methylation are often recruited by members of the methyl-CpG binding domain protein (MBD) family in the nucleus to facilitate the direct transcriptional inhibitory effect of MBD^11^. Moreover, MBD can also associate with transcriptional corepressors such as members of the histone deacetylase (HDAC) family^12^. HDACs act mainly as deacetylation modifiers, removing acetyl groups from chromatin and thus inducing chromatin compaction, which renders it unsuitable for transcription^13^. The HDAC protein family contains 18 proteins that are currently grouped into classes I-IV based on their homology to the corresponding direct homologues in yeast^14^. Recent studies have shown that HDAC proteins are aberrantly expressed in tumour tissues and play an important role in the mechanisms of tumourigenesis and progression^14^, ^15^. Histone deacetylase 7 (HDAC7), a member of the HDAC IIa subfamily, has been reported to deacetylate both histone and nonhistone proteins^16^. High levels of HDAC7 have been shown to correlate with tumour progression and poor prognosis in breast cancer and lung cancer^17^. Mechanistically, HDAC7 has been reported to inhibit Stat3 activation via its deacetylation and to promote lung tumourigenesis^18^. Our previous study in esophageal squamous cell carcinoma (ESCC) showed that HDAC7 overexpression promoted ESCC cell growth and c-Myc expression^17^. As HDAC7 is an important tumourigenic molecule, its clinical significance and potential molecular mechanisms in LUAD deserve further exploration.

In the present study, we investigated the role of DNMT3a in LUAD cell proliferation and metastasis. We found that DNMT3a acts as an oncogenic protein and induces epithelial-to-mesenchymal transition (EMT) in LUAD cells. Overexpression of DNMT3a induces an increase in the expression of ZEB1, a major element of the transcription factor network controlling EMT^19^, by upregulating the expression of HDAC7. Similarly, we found that the oncogenic proliferative role of HDAC7 in LUAD was dependent on expression of c-Myc. Moreover, this study showed that high expression of DNMT3a/HDAC7 was associated with poor prognosis in LUAD patients. Therefore, we propose that DNMT3a can exert oncogenic effects in LUAD by upregulating HDAC7 and further inducing the upregulation of ZEB1 and c-Myc. These results may provide a theoretical and experimental basis for further development of LUAD treatment.

## 2. Materials and Methods

### Patients and materials

The 119 LUAD patients included in this retrospective study had not previously received radiotherapy, chemotherapy, or targeted therapy. The patients received diagnosis and surgical treatment at the Department of Thoracic Surgery, Tangdu Hospital of Air Force Medical University (Xi'an, China), between May 2009 and January 2014. The study was approved by the Ethics Committee. Written informed consent was obtained from all patients before any study-related procedures began. The complete follow-up was updated until death or January 2019, which ever came first. Detailed screening criteria and follow-up content are provided in the Supplementary Information.

### Tissue samples of LUAD patients, tissue microarray, and immunohistochemistry

The 119 pairs of formalin-fixed LUAD tissues and corresponding tumour-adjacent normal tissues were placed in a paraffin-embedded tissue microarray. According to standard practice, immunohistochemical (IHC) staining of paraffin-embedded tissue microarray sections was performed using primary antibodies of anti-DNMT3a (1:400, 20954-1-AP, Proteintech) and anti-HDAC7 (1:100, #33418, Cell Signaling Technology (CST)). In subcutaneous tumour tissue samples from nude mice primary antibodies of anti-DNMT3a (1:800, 20954-1-AP, Proteintech) and anti-HDAC7 (1:1500, 67447-1-lg, Proteintech), anti-c-Myc (1:1000, 26207-1-AP, Proteintech), anti-ZEB1 (1:1000, 21544-1-AP, Proteintech) were used in IHC staining of mice tissues. The scoring of the IHC intensity included negative (score 0), weak (score 1), moderate (score 2), or strong staining (score 3). The proportion of positive stains was scored as 0 (< 5%), 1 (6%-25%), 2 (26%-50%), 3 (51%-75%), or 4 (> 75%). Multiply these 2 scores together to produce the total score. The IHC staining was read by 2 specialist pathologists. Resolve any disagreement between them by discussing with a third pathologist. All pathologists were blinded with no information of the clinical data until statistical analysis. IHC staining results with low or high levels of DNMT3a or HDAC7 expression were stratified by their respective average scores.

### Cell culture and lentivirus infection

The human LUAD cell line (A549, SK-LU-1, H1975) was obtained from the American Type Culture Collection (ATCC) and cultured in Dulbecco's Modified Eagle Medium (DMEM; Gibco), supplemented with 10% fetal bovine serum (FBS, Gibco, NY, USA), 1% (v/v) penicillin-streptomycin solution (Hyclone). The DNMT3a, HDAC7, ZEB1 and corresponding control lentiviruses were obtained from Genechem Corporation (Shanghai, China). Lentiviral infection of A549, SK-LU-1 and H1975 cells was performed according to the protocol of Genechem's Recombinant Lentivirus Operations Manual, and further stable cell lines were selected.

### 5‑Ethynyl‑2′‑deoxyuridine incorporation assay

Cell proliferation was assessed by 5‑Ethynyl‑2′‑deoxyuridine (EdU) incorporation assay. Use the BeyoClick EdU cell proliferation kit with Alexa Fluor 594 (Beyotime, Shanghai, China) according to the manufacturer's instructions. Cells were imaged by Olympus FV1000 confocal microscope (Olympus, Tokyo, Japan). The EdU positive cells were manually counted and expressed as the percentage of cells calculated from nuclear labeling with Hoechst 33342.

### Colony formation assay

4-8×10^2^ cells were seeded and cultured in DMEM containing 10% FBS or 5% FBS with cell treatment agents in 6-well plates for 10-14 days for clonal formation experiments. The colonies were then fixed with formalin and stained with 0.1% crystal violet. The plates were photographed and the colonies were counted using the ImageJ software (National Institutes of Health).

### Wound healing assay

Wound healing assays were performed in 6-well plates. Cells were seeded into six-well plates with DMEM containing 10% FBS or 5% FBS. A wound was created by scraping the cell monolayer with a tip of the p200 pipette after the cells reached 90-100% confluence. Then, each well was washed three times with phosphate buffered saline (PBS) and cultured in serum-free media (SFM) with or without cell treatment agents. Digital images were taken with an inverted microscope immediately after scratching and 48 hours after scratching. The ImageJ software was used to calculate the cell-free scratch area immediately after the scratch and the cell-free remaining area at the end of the measurement.

### Transwell cell migration assay

The abilities of cell migration were quantified by transwell assays. Cell transwell chambers were obtained from Corning (Costar 3422). Cells were seeded on the upper chamber and filled with 200 μl SFM, and the lower chamber was filled with 500 μl DMEM containing 10% FBS. After 24 hours, the migrating cells were immobilized with formalin, stained with 0.1% crystal violet, photographed and counted.

### RNA sequencing and pathway enrichment analysis

Total RNA was extracted from the control and DNMT3a knockdown groups of SK-LU-1 cells using TRIzol reagent (Invitrogen Corporation). Further RNA sequencing (RNA-seq) detection and analysis were conducted using BGISEQ-500 platform (BGI Corporation). The pathway analysis for differentially expressed genes (DEGs) was performed based on the Gene Ontology (GO) database. The Dr. Tom online platform of BGI was used for data analysis (http:// report. bgi.com).

### Quantitative real‑time polymerase chain reaction

Total RNA was harvested from cells with TRIzol reagent (Invitrogen Corporation). Complementary DNA was generated using a Prime Script RT Master Mix (TaKaRa). Quantitative real-time polymerase chain reaction (qRT-PCR) was performed with SYBR Premix Ex Taq II (TaKaRa) to detect targeted messenger RNA (mRNA) levels, and data were analyzed with the MxPro software (Stratagene). GAPDH expression was used as an internal control. The primer sequences involved in qRT-PCR are as follows: ZEB1, forward GATGATGAATGCGAGTCAGATGC, reverse ACAGCAGTGTCTTGTTGTTGT; GAPDH, forward TGACTTCAACAGCGACACCCA, reverse CACCCTGTTGCTG TAGCCAAA.

### Western blotting

Western blotting was performed according to the procedures described previously^20^, loading 30 μg of total protein lysate per lane. The following primary antibodies were used: anti-DNMT3a (1:1000, 20954-1-AP, Proteintech), anti-HDAC7 (1:1000, 67447-1-lg, Proteintech), anti-c-Myc (1:1000, 26207-1-AP, Proteintech), anti-E-cadherin (1:1000, #14472, CST), anti-ZEB1 (1:1000, 21544-1-AP, Proteintech), anti-Flag tag (1:2000, AE063, ABclonal), anti-CDK6 (1:1000,14052-1-AP, Proteintech), anti-CDK4 (11026-1-AP, Proteintech), anti-Cyclin D1(1:5000,60186-1-Ig, Proteintech) and anti-β-actin (1:1000, #3700, CST). The secondary antibodies used were HRP-linked anti-IgG antibodies (1:5000, Zhongshan Company).

### Cell treatment

SGI1027 (HY-13962, MedChem Express), SAHA (HY-10221, MedChem Express) and TMP269 (HY-18360, MedChem Express) stock solutions were prepared in DMSO media prior to the subsequent experiments. The LUAD cells were treated with these chemical compounds for 48h and then harvested for further analyses.

### Animal experiments

All animal experiments were approved by the Animal Care Committee of PLA General Hospital. Male athymic nude mice (4-6 weeks, 16-18 g) were obtained from the Laboratory Animal Center of PLA General Hospital. SGI1027 (S873364, Macklin), SAHA (S830534, Macklin) and TMP269 (T860915, Macklin) were used to treat nude mice. Nude mice were intraperitoneally injected with PBS, SGI1027 (15mg/kg, once every two days, 16 days), SAHA (50mg/kg, once every two days, 16 days) and TMP269 (40mg/kg, once every two days, 16 days) two weeks after subcutaneous tumour vaccination respectively. Mice were kept under a 12-h light-dark cycle. Temperatures of 65-75 °F (18-23 °C) with 40-60% humidity were also used as housing conditions for the mice.

### *In vivo* tumour xenograft assay and lung metastasis assay

Nude mice were randomly divided into groups (5 mice per group) and received respective treatments. Briefly, for *in vivo* xenograft assay, different groups of cells (5×10^6^ in 200 µl PBS) were separately and subcutaneously inoculated into the nude mice. The body weight of each mouse was measured every 3 days for 21-31 days. Tumours were then excised from the sacrificed mice for additional analysis.

For the tail vein metastasis assay, 5×10^6^ cells resuspended in 100 µl of PBS were injected into the lateral tail vein of nude mice. After 4 weeks, their lungs were dissected and prepared for standard histological examination.

### Immunoprecipitation (IP)

The normal immunoglobin G (IgG, Millipore) was performed as the negative control. For the exogenous IP, per 1 mg whole cell lysates were incubated with 8 μg anti- Flag agarose gel (A-2220, sigma) at 4 °C for 4 h. For the endogenous IP, the lysates were incubated with 8 μg indicated immunoprecipitation antibody overnight at 4 °C, then further incubated with 40 μl PuroProteome Protein G Magnetic Beads (LSKMAGG02, Millipore) for 2 h. Later, the immunocomplexes were washed 4 times with NETN buffer, then diluted with the loading buffer and boiled for further immunoblotting.

### Statistical analysis

The quantitative data were compared between groups using the t-test. Categorical data were analyzed using the χ2-test or Fisher's exact test. The overall survival rates were determined using the Kaplan-Meier method and log-rank test. Univariate or multivariate survival analysis was carried out using the Cox proportional hazards model. A value of *p* < 0.05 was considered to be significant. SPSS 29.0 software (IBM Corp) was used to analyze the data.

## 3. Results

### DNMT3a is upregulated in LUAD, and high expression of DNMT3a predicts poor prognosis and survival in LUAD patients

**Figure 1:**
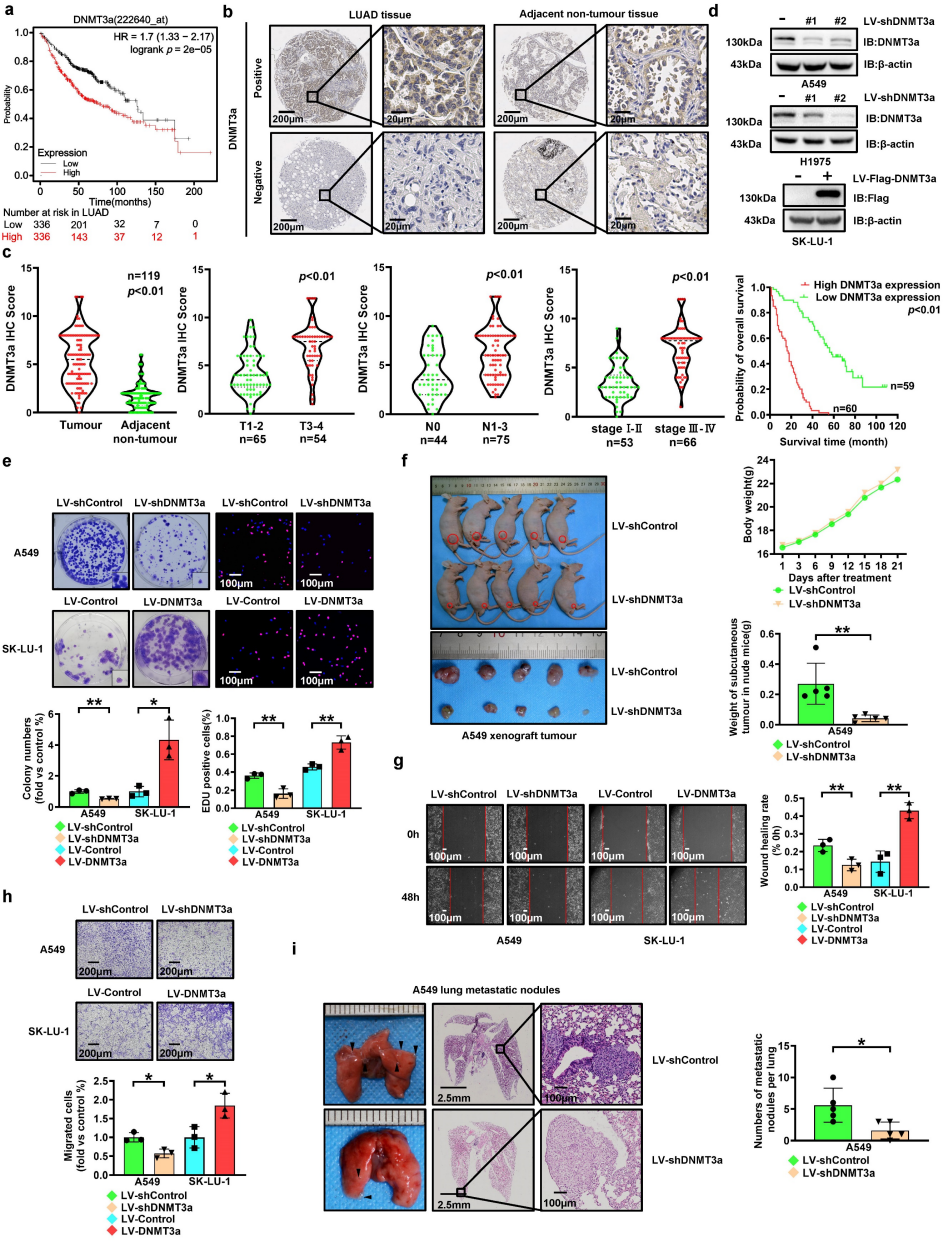
** High expression of DNMT3a predicts poor prognosis in LUAD. a** Kaplan-Meier survival analysis of patients represented in the Kaplan-Meier Plotter database stratified by DNMT3a expression. **b** Representative images of DNMT3a IHC staining in LUAD and adjacent nontumour tissues. Scale bars, 200 μm and 20 μm (inset). **c** Statistical analysis of DNMT3a expression based on IHC staining in tumour tissue and adjacent nontumour tissue of 119 LUAD patients. Statistical analysis of DNMT3a expression based on IHC staining of samples from 119 LUAD patients stratified into subgroups of T classification, N classification and clinical stage. Kaplan-Meier survival analysis of 119 LUAD patients stratified by DNMT3a expression. **d** Representative results of western blot analysis of DNMT3a and Flag expression in DNMT3a knockdown and DNMT3a overexpression LUAD cells. β-actin was used as an internal control. **e** Representative images and statistical analysis of the colony formation assay. Colonies were visualized by crystal violet staining. Representative images and statistical analysis of the EdU incorporation assay. The results were calculated as the ratio of the number of EdU-positive cells (red fluorescence) to the total number of Hoechst 33342-positive cells (blue fluorescence). Scale bar, 100 μm (inset). **f** Gross photograph of subcutaneous xenograft tumours and data showing the changes in subcutaneous tumour weight and nude mouse body weight in each group. **g** Representative wound healing assay images and results. The migration ability was quantified as the mean scratch area at each time point. The initial scratch area (0 h) was set as 100%. Scale bars, 100 μm (inset). h Representative transwell migration assay images and results. Scale bars, 200 μm (inset). **i** Representative gross photographs and HE staining images of lung samples from the indicated groups. Scale bars, 2.5 mm and 100 μm (inset). Numbers of metastatic nodules per lung in the HE staining images from each group. ^*^ p < 0.05. ^**^ p < 0.01.

To investigate the expression of DNMT3a, 672 LUAD cases represented in the Kaplan‒Meier Plotter database (http://KMplot.com) were analysed. Unexpectedly, high expression of DNMT3a was significantly correlated with poor survival in these LUAD patients (Fig. 1a). In order to validate this finding, we analysed the expression of the DNMT3a protein and the clinical significance of DNMT3a by IHC staining (Fig. 1b and c) in a paraffin-embedded tissue microarray containing 119 paired tumour and normal tissue samples. According to the average IHC score of DNMT3a, LUAD samples and paired adjacent normal tissue samples were divided into high expression group and low expression group of DNMT3a. DNMT3a levels were significantly increased in LUAD tissues compared to adjacent normal tissues (Fig. 1b and c). Furthermore, LUAD patients with deep tumour invasion and/or a large tumour size (T3/T4), a high American Joint Committee on Cancer (AJCC) 8th edition stage (stage III-IV) and lymph node metastasis (N1-N3) had higher expression of DNMT3a (Fig. 1c). Upregulated expression of DNMT3a was positively correlated with maximal tumour size, high T stage, lymphatic invasion, high AJCC 8th edition stage, and poor tumour differentiation (Table 1). Kaplan‒Meier analysis showed that high expression of DNMT3a was correlated with poor overall survival in LUAD patients (Fig. 1c). Cox regression analyses showed that DNMT3a was an independent prognostic factor for LUAD patients (HR=7.887, 95% CI 4.724-13.169, *p* < 0.01; HR=2.699, 95% CI 1.372-5.311, *p* < 0.01, Table 2).

### DNMT3a promotes proliferation and metastasis of LUAD

To investigate the role of DNMT3a in malignant progression of LUAD, we performed lentiviral transduction to establish LUAD cell lines with DNMT3a knockdown or overexpression based on the basal DNMT3a levels in LUAD cell lines (Fig. 1d). Compared with that in the respective control groups, DNMT3a knockdown markedly inhibited the clonogenic ability of cells, and DNMT3a overexpression markedly enhanced the clonogenic ability of cells, as determined by a colony formation assay (Fig. 1e). Moreover, the proliferation ability was reduced in the DNMT3a knockdown cell line, while the DNMT3a overexpression cell lines showed the opposite trend, as demonstrated by the number of EdU-positive cells detected in the EdU incorporation assay (Fig. 1e). To further verify the function of DNMT3a in promoting LUAD cell proliferation *in vivo*, we performed xenograft assays in nude mice. Consistent with the results *in vitro*, the mean weight of subcutaneous tumours in the shDNMT3a group was observably lower than that in the control group (Fig. 1f).

The metastatic ability was also modulated by the DNMT3a. Specifically, DNMT3a knockdown significantly suppressed cell migration, as demonstrated by the wound healing assay and transwell assay, suggesting that DNMT3a knockdown inhibited migration in these cells (Fig. 1g and h); however, a trend towards enhanced migration was observed in the DNMT3a overexpression group (Fig. 1g and h). *In vivo*, downregulation of DNMT3a observably reduced the incidence of lung metastasis, as shown by the number of metastatic lung nodules formed in the tail vein metastasis assay in nude mice (Fig. 1i).

### The expressions of c-Myc and ZEB1 are upregulated by DNMT3a via increasing HDAC7 in LUAD cell lines

**Figure 2:**
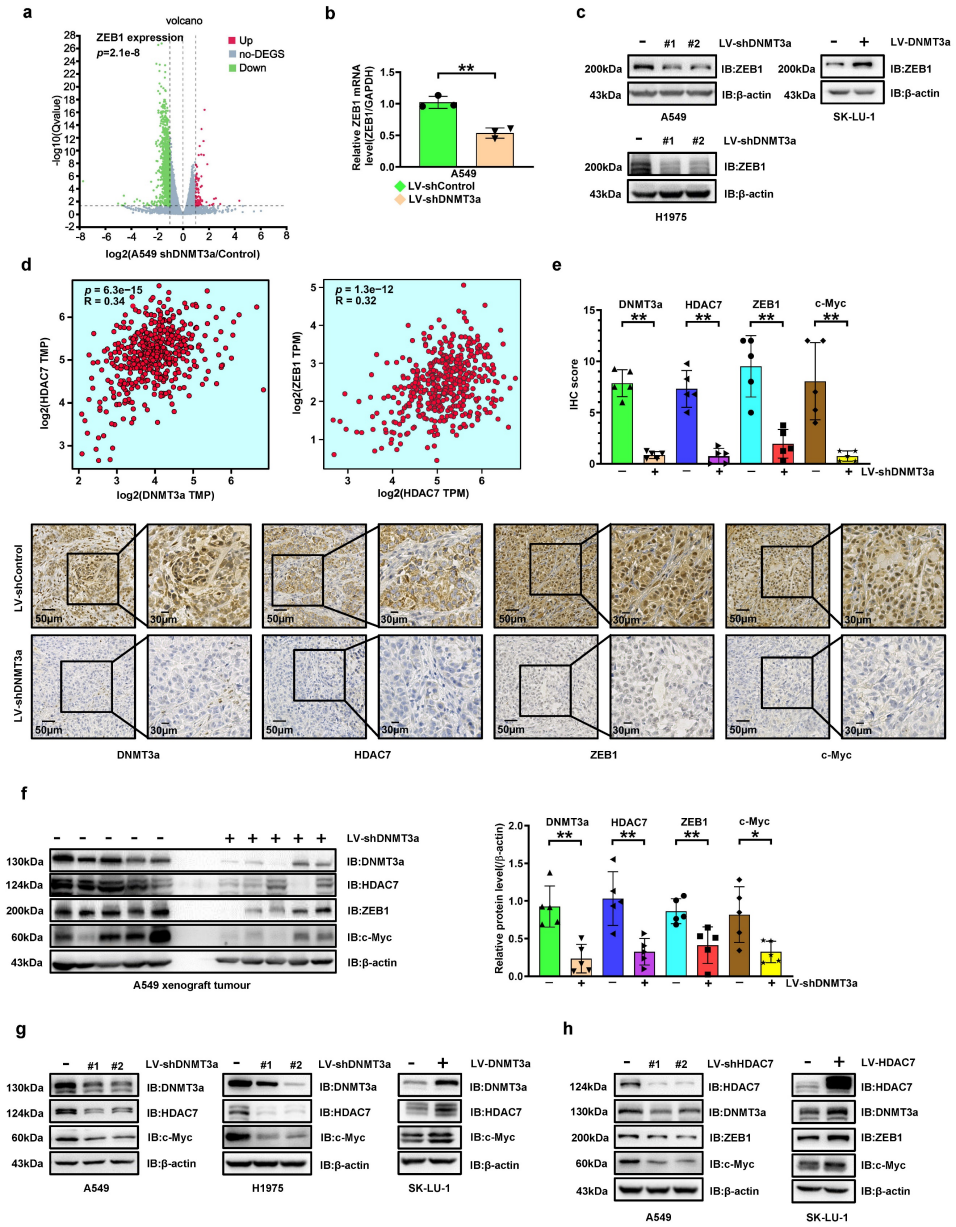
** DNMT3a knockdown inhibits HDAC7 expression, thereby decreasing the expression of ZEB1 and c-Myc in LUAD. a** The volcano plot of differentially expressed genes (DEGs) identified by RNA-seq analysis of DNMT3a knockdown cells and control cells. Red represents upregulated DEGs, green represents downregulated DEGs, and gray represents non-DEG. **b** Statistical analysis of relative ZEB1 mRNA levels in DNMT3a knockdown cells and control cells. Target mRNA expression was normalized to GAPDH mRNA expression. **c** Representative results of western blot analysis of ZEB1 in DNMT3a knockdown and DNMT3a overexpression LUAD cells. β-actin was used as an internal control. **d** Gene expression profiling interactive analysis based on TCGA data to evaluate the relationships between DNMT3a and HDAC7 and between HDAC7 and ZEB1 in LUAD. **e** Representative images and statistical analysis of DNMT3a, HDAC7, ZEB1 and c-Myc IHC staining in xenograft tumours from nude mice in the shDNMT3a group and the control group. Scale bars, 50 μm and 30 μm (inset). **f** Representative results of western blot analysis of DNMT3a, HDAC7, ZEB1 and c-Myc expression in xenograft tumour cells from nude mice in the shDNMT3a group and the control group. β-actin was used as an internal control. **g** Representative results of western blot of DNMT3a, HDAC7, and c-Myc expression in DNMT3a knockdown and DNMT3a overexpression LUAD cells. β-actin was used as an internal control. **h** Representative results of western blot of HDAC7, DNMT3a, ZEB1, and c-Myc expression in DNMT3a knockdown and DNMT3a overexpression LUAD cells. β-actin was used as an internal control. ^*^ p < 0.05.^ **^ p < 0.01.

To explore the potential mechanism by which DNMT3a enhances LUAD cells proliferation and metastasis, RNA-seq-based transcriptome analysis was performed in A549 cells to compare transcriptome changes between the control and shDNMT3a groups. Among the differential genes associated with EMT, ZEB1, an EMT biomarker, attracted our attention in the volcano plot (Fig. 2a). Trends consistent with the above results were confirmed by qRT‒PCR, which showed that the mRNA expressions of ZEB1 was ere downregulated in shDNMT3a A549 and H1975 cells (Fig. 2b). And western blotting showed that ZEB1 protein expression in shDNMT3a A549 cells was lower than that in control cells, and higher in DNMT3a overexpression SK-LU-1 cells (Fig. 2c). ZEB1 expression has been detected in numerous solid tumours and found to be significantly correlated with poor prognosis^19^. However, the relationship between DNMT3a and ZEB1 was unclear. Thus, we carried out gene expression profiling interactive analysis (http://gepia2.cancer-pku.cn/#index) with data from The Cancer Genome Atlas (TCGA) datasets and found that DNMT3a expression was positively correlated with HDAC7 expression and that HDAC7 expression was positively correlated with ZEB1 expression in LUAD (Fig. 2d). In our previous study, we found that c-Myc was upregulated via overexpression of HDAC7, thereby facilitating the escape of tumour cells from senescence and promotes their growth^17^, which was also confirmed in LUAD cells in the present study.

In order to verify the regulatory relationship between DNMT3a and HDAC7, ZEB1, c-Myc and their impact on LUAD proliferation, we analysed the protein expression of DNMT3a, HDAC7, ZEB1 and c-Myc by IHC staining in xenografts from nude mice in the shDNMT3a group and the corresponding control group. Compared with those in the control group, the protein expression levels of DNMT3a, HDAC7, ZEB1 and c-Myc were simultaneously decreased (Fig. 2e). The same trend was confirmed by western blot analysis of xenografts from nude mice (Fig. 2f).

The significant decreases in HDAC7 and c-Myc protein levels in DNMT3a knockdown LUAD cells were validated by western blotting (Fig. 2g). Furthermore, overexpression of DNMT3a significantly upregulated the expression of HDAC7 (Fig. 2g and S1), c-Myc (Fig. 2g and S7), and ZEB1 protein (Fig. 2c and S7). To further confirm the mechanism linking DNMT3a and HDAC7, we established LUAD cell lines with HDAC7 knockdown and overexpression by lentiviral transduction (Fig. 2h). The characteristics of LUAD cells with modulation of HDAC7 protein expression were highly consistent with those of LUAD cells with modulation of DNMT3a protein expression: the ZEB1 and c-Myc protein levels were significantly decreased in HDAC7 knockdown LUAD cells. Furthermore, overexpression of HDAC7 significantly upregulated the protein expression of ZEB1 and c-Myc (Fig. 2h). Moreover, HDAC7 had a positive effect on the DNMT3a protein level in these cells (Fig. 2h), forming a positive feedback loop with DNMT3a.

Besides, except for upregulating the ZEB1 and c-Myc expression, overexpression of DNMT3a or HDAC7 can downregulate E-cadherin and upregulate CDK6, and vice versa (Fig. S2a and b). But DNMT3a had no effect on the protein expression levels of CDK4 and cyclin Dl (Fig. S2a). Immunoprecipitation was performed to demonstrate the absence of an interaction between ZEB1 and HDAC7 (Fig. S8a), and western blot analysis demonstrated that knockdown of ZEB1 in LUAD cells did not affect the expression of HDAC7 and c-Myc, but significantly upregulated the expression of E-cadherin (Fig. S8b).

### HDAC7 is upregulated in LUAD, and high expression of HDAC7 predicts poor prognosis in LUAD patients

**Figure 3:**
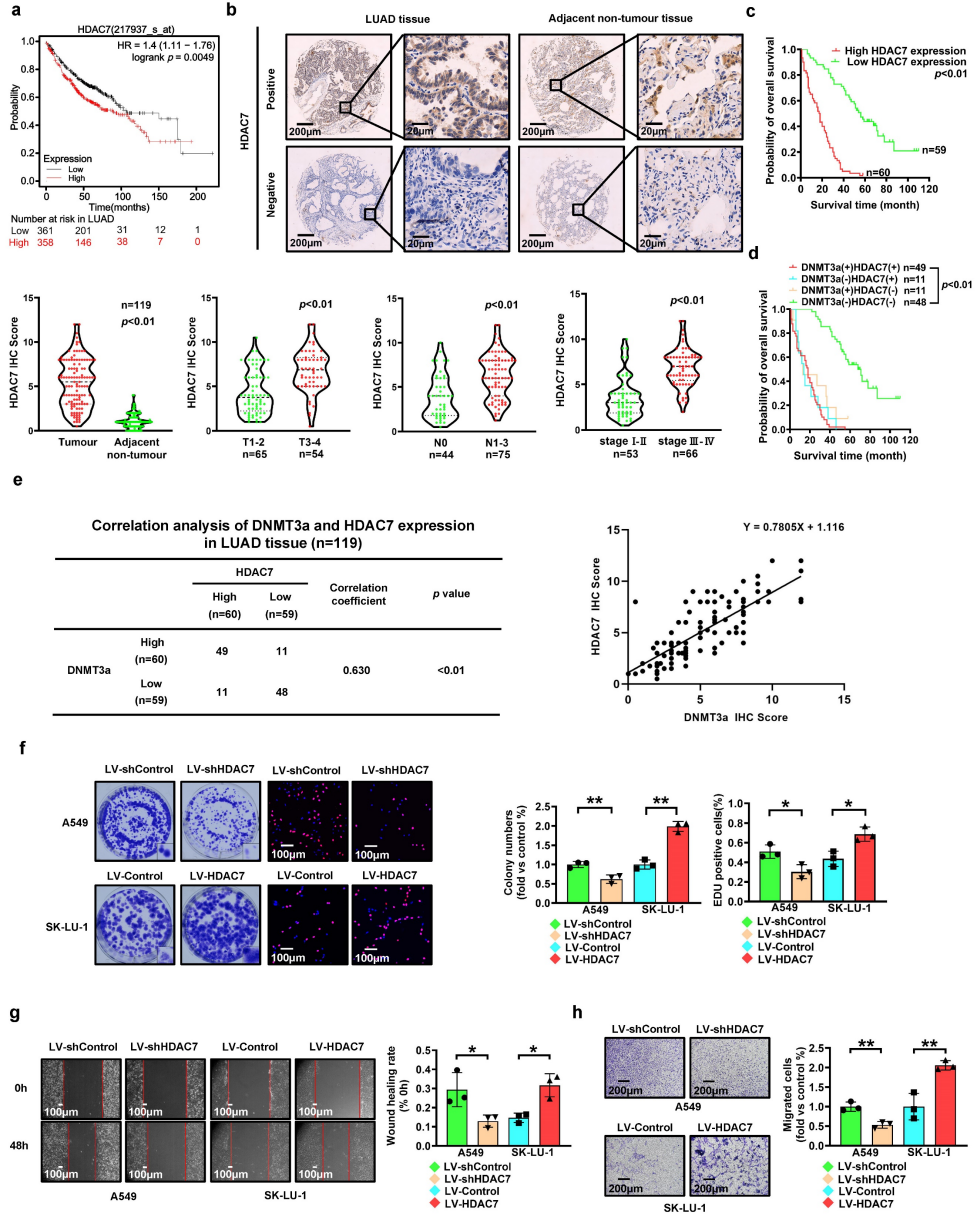
** High expression of HDAC7 predicts poor prognosis in LUAD. a** Kaplan-Meier survival analysis of patients represented in the Kaplan-Meier Plotter database stratified by HDAC7 expression. **b** Representative images of HDAC7 IHC staining in LUAD and adjacent nontumour tissues. Scale bars, 200 μm and 20 μm (inset). **c** Statistical analysis of HDAC7 expression based on IHC staining in tumour tissue and adjacent nontumour tissue of 119 LUAD patients. Statistical analysis of HDAC7 expression based on IHC staining of samples from 119 LUAD patients stratified into subgroups of T classification, N classification and clinical stage. Kaplan-Meier survival analysis of 119 LUAD patients based on HDAC7 expression. **d** Comprehensive Kaplan-Meier survival analysis of 119 LUAD patients stratified by DNMT3a and HDAC7 expression based on tissue microarray IHC results. **e** Correlation analysis of DNMT3a and HDAC7 expression in LUAD tissues. **f** Representative images and statistical analysis of the colony formation assay. Colonies were visualized by crystal violet staining. Representative images and statistical analysis of the EdU incorporation assay. The results were calculated as the ratio of the number of EdU-positive cells (red fluorescence) to the total number of Hoechst 33342-positive cells (blue fluorescence). Scale bar, 100 μm (inset). **g** Representative wound healing assay images and results. The migration ability was quantified as the mean scratch area at each time point. The initial scratch area (0 h) was set as 100%. Scale bars, 100 μm (inset). **h** Representative transwell migration assay images and results. Scale bars, 200 μm (inset). ^*^ p < 0.05.^ **^ p < 0.01.

To investigate the expression of HDAC7, 719 LUAD cases in the Kaplan‒Meier Plotter database (http://KMplot.com) were analysed. High expression of HDAC7 was significantly correlated with poor survival in this cohort of LUAD patients (Fig. 3a). To verify this finding, we analysed HDAC7 protein expression and the clinical significance of HDAC7 by IHC staining in a paraffin-embedded tissue microarray containing 119 paired tumour and normal tissue samples. According to the average IHC score of HDAC7, LUAD samples and paired adjacent normal tissue samples were divided into high expression group and low expression group of HDAC7.

The HDAC7 levels were significantly increased in the LUAD tissues compared to those in the adjacent normal tissues (Fig. 3b). Furthermore, LUAD patients with deep tumour invasion and/or a high T stage (T3/T4), a high American Joint Committee on Cancer (AJCC) 8th edition stage (stage III-IV) and lymph node metastasis (N1-N3) had higher expression levels of HDAC7 (Fig. 3b). In addition, upregulated expression of HDAC7 was positively correlated with high T stage, lymphatic invasion, high TNM stage, and poor tumour differentiation (Table 1). Kaplan-Meier analysis showed that high expression of HDAC7 was correlated with poor overall survival in LUAD patients (Fig. 3c). Moreover, Cox regression analyses showed that HDAC7 was an independent prognostic factor for LUAD patients (HR=5.967, 95% CI 3.694-9.639, *p* < 0.01; HR=2.247, 95% CI 1.238-4.078, *p* < 0.01, Table 2).

### Correlation of DNMT3a and HDAC7 expression with prognosis in LUAD patients

Correlation analysis revealed a significant correlation between DNMT3a and HDAC7 expression in LUAD tissues (correlation coefficient=0.630, *p* < 0.01, Fig. 3e). Patients were divided into four groups according to the DNMT3a and HDAC7 levels. The Kaplan-Meier analyses suggested that LUAD patients with low DNMT3a/low HDAC7 expression had longer overall survival time (Fig. 3d).

### HDAC7 promotes proliferation and metastasis of LUAD

To investigate the role of HDAC7 in LUAD, we used lentiviral transduction to establish LUAD cell lines with HDAC7 knockdown and overexpression based on the basal HDAC7 levels in LUAD cell lines (Fig. 2h). Compared with that in the respective control groups, HDAC7 knockdown markedly reduced the clonogenic ability of cells, whereas HDAC7 overexpression markedly enhanced this ability, as determined by the colony formation assay (Fig. 3f). Moreover, the proliferation ability was reduced in the HDAC7 knockdown cell lines, while the HDAC7 overexpression cell lines showed the opposite trend, as demonstrated by the number of EdU-positive cells detected in the EdU incorporation assay (Fig. 3f).

The metastasis ability was also modulated by regulation of HDAC7 expression. Knockdown of HDAC7 significantly weakened cell migration, as demonstrated by the wound healing assay and transwell assay, suggesting that HDAC7 knockdown inhibited migration ability in these cells (Fig. 3g and h); however, a trend towards enhanced migration was observed in the HDAC7 overexpression group (Fig. 3g and h).

### DNMT3a promotes cell proliferation and metastasis via HDAC7 signalling pathway in LUAD

**Figure 4:**
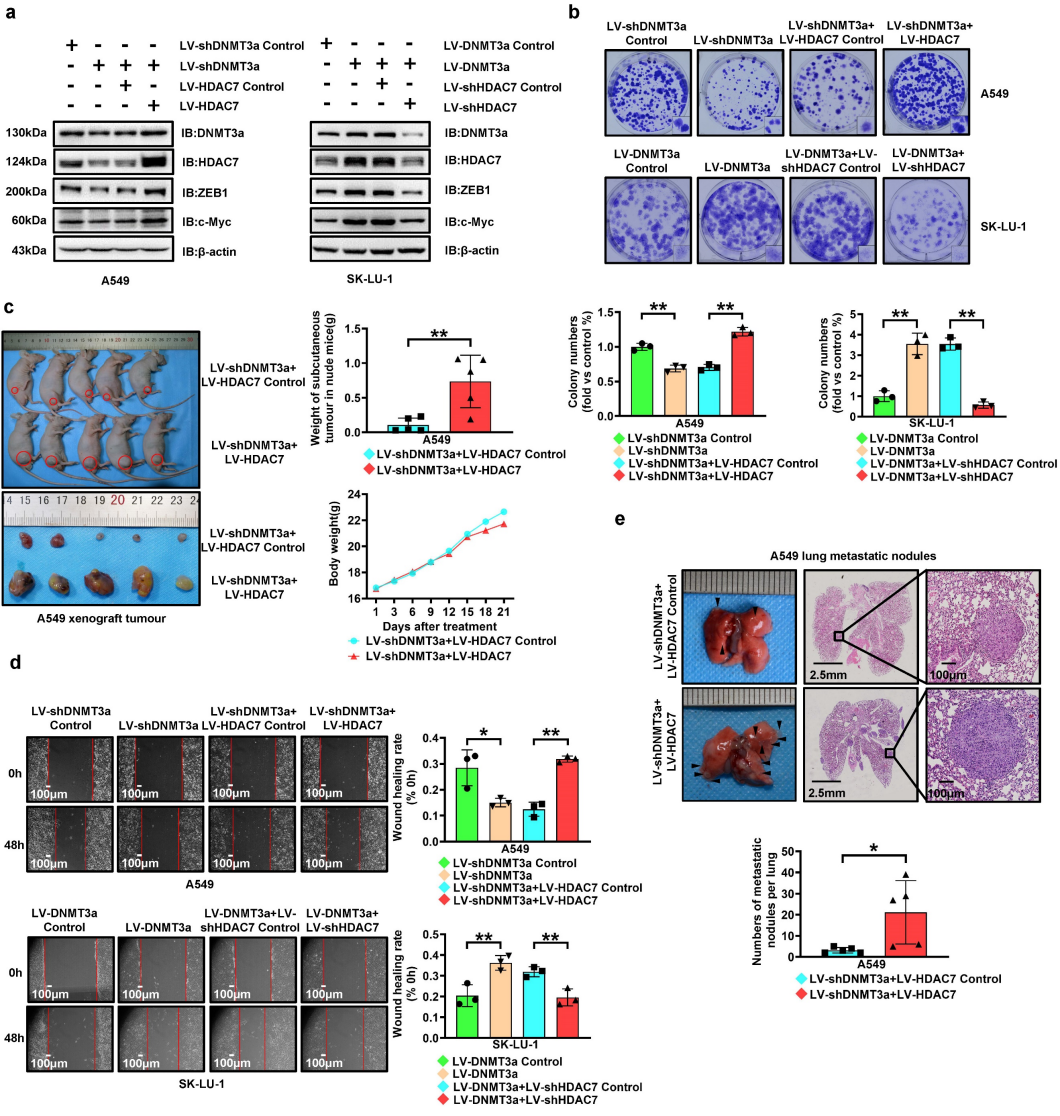
** DNMT3a promotes cell proliferation and metastasis via upregulating HDAC7 in LUAD. a** Representative western blot analysis of DNMT3a, HDAC7, ZEB1 and c-Myc expression in the indicated groups. β-actin was used as an internal control. **b** Representative images and statistical analysis of the colony formation assay. Colonies were visualized by crystal violet staining. **c** Gross photograph of subcutaneous xenograft tumours and data showing the changes in subcutaneous tumour weight and nude mouse body weight in the indicated groups. **d** Representative wound healing assay images and results. The migration ability was quantified as the mean scratch area at each time point. The initial scratch area (0 h) was set as 100%. Scale bars, 100 μm (inset). **e** Representative gross photographs and HE staining images of lung samples from the indicated groups. Scale bars, 2.5 mm and 100 μm (inset). Numbers of metastatic nodules per lung in the HE staining images from each group. ^*^ p < 0.05.^ **^ p < 0.01.

To further confirm the involvement of HDAC7 in DNMT3a-regulated LUAD progression, we upregulated HDAC7 in DNMT3a knockdown A549 cells and downregulated HDAC7 in DNMT3a overexpression SK-LU-1 cells by lentiviral transduction. Western blotting not only showed that either overexpression or knockdown of HDAC7 partially reversed the DNMT3a-induced changes in the expression of proteins, including ZEB1, c-Myc, E-cadherin, and CDK6, but also confirmed the existence of positive feedback regulation between DNMT3a and HDAC7 (Fig. 4a and S2c).

HDAC7 overexpression partially reversed the reduction in the clonogenic ability resulting from DNMT3a knockdown, as shown by the colony formation assay (Fig. 4b). Similarly, HDAC7 knockdown partially attenuated the increase in the proliferative ability resulting from DNMT3a overexpression (Fig. 4b). *In vivo*, HDAC7 overexpression was verified to significantly increase the weight of subcutaneous tumours in the A549 DNMT3a knockdown group, as demonstrated by xenograft assays in nude mice (Fig. 4c). It was confirmed by IHC that compared with the LV-shDNMT3a+LV-HDAC7 Control group, the protein expression levels of DNMT3a, ZEB1 and c-Myc were upregulated in the group of LV-shDNMT3a+LV-HDAC7 (Fig. S3a). The same trend was verified by western blot analysis of xenografts from nude mice (Fig. S3b).

To investigate the migration ability, a wound healing assays was adopted, and the results revealed that HDAC7 overexpression significantly enhanced the migration ability of DNMT3a knockdown A549 cells, whereas the DNMT3a-induced enhancement of the migration ability was attenuated by HDAC7 knockdown (Fig. 4d). *In vivo*, HDAC7 overexpression was confirmed to markedly increase the number of lung metastatic nodules in the A549 DNMT3a knockdown group, as determined by the tail vein metastasis assay in nude mice (Fig. 4e).

### Inhibition of DNMT3a and/or HDAC7 inhibits DNMT3a-induced malignant progression of LUAD

**Figure 5:**
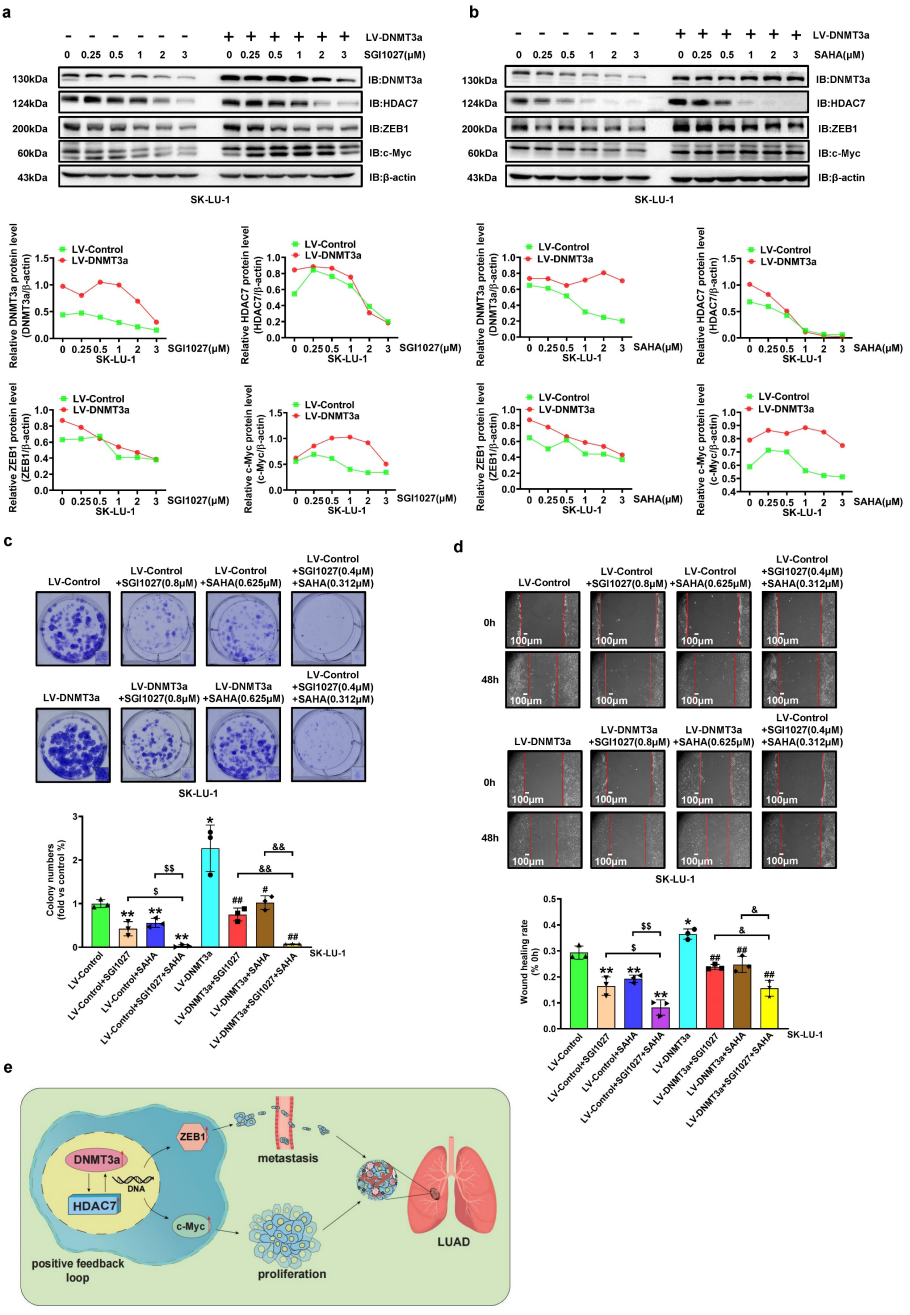
** DNMT3a and HDAC7 formed a positive feedback loop promotes LUAD cell progression. a** Representative western blot and statistical analysis of DNMT3a, HDAC7, ZEB1 and c-Myc expression in DNMT3a overexpression LUAD cells and control cells after treatment with SGI1027 for 48 h. β-actin was used as an internal control. **b** Representative western blot and statistical analysis of DNMT3a, HDAC7, ZEB1 and c-Myc expression in DNMT3a-overexpression LUAD cells and control cells after treatment with SAHA for 48 h. β-actin was used as an internal control. **c** Representative images and statistical analysis of the colony formation assay in the indicated groups after treatment with SGI1027 and/or SAHA for 48 h. Colonies were visualized by crystal violet staining. **d** Representative wound healing assay images and statistical analysis in the indicated groups after treatment with SGI1027 and/or SAHA for 48 h. The migration ability was quantified as the mean scratch area at each time point. The initial scratch area (0 h) was set as 100%. Scale bars, 100 μm (inset).** e** Schematic diagram of the mechanism by which DNMT3a promotes tumour proliferation and metastasis by upregulating HDAC7 and further inducing the upregulation of ZEB1 and c-Myc in LUAD. ^*^ p < 0.05 vs. the LV-Control group,^**^ p < 0.01 vs. the LV-Control group,^ ##^* p* < 0.01 vs. the LV-DNMT3a group, ^$$^*p* < 0.01 vs. the LV-Control+SGI1027+SAHA group, ^&&^* p* < 0.01 vs. the LV-DNMT3a+ SGI1027+SAHA group.

To further confirm that HDAC7 participates in the DNMT3a-mediated malignant progression of LUAD, a DNMT3a inhibitor (SGI1027), HDAC7 inhibitor (SAHA) and HDAC7 specific inhibitor (TMP269) were used. DNMT3a overexpression SK-LU-1 cells and the corresponding control cells were treated with SGI1027, SAHA and TMP269 at various concentrations for 48 hours. The alterations in DNMT3a, HDAC7, c-Myc, and ZEB1 expression induced by DNMT3a overexpression were inhibited by SGI1027, SAHA or TMP269 treatment in a dose-dependent manner (Fig. 5a, 5b and S4a). Moreover, the existence of positive feedback regulation between DNMT3a and HDAC7 was further confirmed. The colony formation assay and wound healing assay demonstrated that the combination of SGI1027 and SAHA inhibited cell proliferation and metastasis better than either SGI1027 or SAHA alone in LUAD (Fig. 5c and d). The same result can be seen in cells treated with SGI1027 and TMP269 (Fig. S4b and S4c).

*In vivo*, SGI1027, SAHA and TMP269 were used to treat nude mice implanted with subcutaneous tumours and reveal that the mean weight of subcutaneous tumours in the SGI1027, SAHA or TMP269 groups were observably lower than that in the control group (Fig S5a). It was confirmed by IHC that compared with the Control group, the protein expression levels of DNMT3a, ZEB1 and c-Myc were downregulated in the groups of SGI1027, SAHA or TMP269 (Fig. S5b). The same trend was verified by western blot analysis of xenografts from nude mice (Fig. S6a, b and c).

## 4. Discussion

LUAD is the most common type of lung cancer, with an annual incidence of more than 1 million cases worldwide^21^. Despite current advances in treatment options, determining approaches to stratify patients and use appropriate therapies to improve patient outcomes remain high-priority challenges. Therefore, the molecular mechanisms underlying malignant progression of LUAD need to be further explored to facilitate the development of therapeutic strategies.

In our analysis of 672 LUAD cases from the Kaplan‒Meier Plotter database, high expression of DNMT3a was significantly associated with poor survival in LUAD patients. In addition, our IHC analysis showed that the expression of DNMT3a in LUAD tissues was significantly higher than that in adjacent non-tumour tissues, and high expression of DNMT3a was positively correlated with high TNM stage and poor tumour differentiation. Furthermore, Kaplan‒Meier survival analysis showed that high expression of DNMT3a was associated with shorter overall survival time in LUAD patients and that patients with low expression of DNMT3a had longer median survival times. Multivariate Cox survival analysis suggested that DNMT3a was an independent negative prognostic factor in LUAD.

DNMT3a is thought to exhibit preferential activity toward unmethylated DNA, resulting in *de novo* DNA methylation, a process by which new methyl groups can be actively transferred to DNA sequences to silence tumour suppressor genes and that plays a crucial role in carcinogenesis^1^, ^22^. Previous studies have reported that genetic mutation or abnormal expression of DNMT3a, which is considered to be a key element in oncogenic epigenetic reprogramming, is closely related to the occurrence and malignant progression of haematological malignancies and solid tumours^23^, ^24^. In acute monocytic leukaemia, DNMT3a, with a high mutation frequency, was found to be associated with poor prognosis and was expected to be a novel molecular marker for diagnostic and prognostic evaluation^25^. Mutation of DNMT3a can affect the DNA methylation levels of leukaemia-related genes, thus exerting carcinogenic effects by blocking haematopoietic cell differentiation and promoting excessive proliferation^26^. The development of AML is induced by conditional knock-in of mutant DNMT3a in the haematopoietic system of mice, and abnormal mTOR pathway activation caused by DNMT3a mutation-mediated hypomethylation is considered a potential therapeutic target^27^. Some studies in solid tumours have reported that DNMT3a is upregulated in ovarian cancer, gastric adenocarcinoma, and bladder cancer and is associated with poor prognosis^28^-^30^. Overexpression or mutation of DNMT3a can cause biological behaviours related to malignant progression, such as the Warburg effect^28^. At the level of DNA epigenetic modification, DNMT3a has been shown to promote colorectal cancer progression and oxaliplatin resistance by directly binding to the promoter regions of DAB2IP and MEIS1^22^, ^31^. Based on these results, the DNMT3a-mediated epigenetic regulation mechanism can be correlated with the occurrence, malignant progression and drug resistance of different tumours and could be a novel prognostic biomarker. Our results suggested that a high level of DNMT3a was associated with a shorter survival time in patients with LUAD, implying the considerable importance of exploring the molecular mechanism by which DNMT3a mediates malignant progression of LUAD.

A member of the HDAC family, HDAC7 is involved in the regulation of cell proliferation, apoptosis, differentiation and migration^18^. We previously studied the oncogenic role of HDAC7 in solid tumours. In ESCC, HDAC7 can upregulate tumour progression mediated by WNT5A, regulate the expression of EMT-related molecules such as SNAIL and E-cadherin, and enhance the migration and invasion abilities of ESCC cells^32^. In choroidal melanoma, HDAC7/c-Myc signalling pathway activity was confirmed to enhance tumour cell proliferation and metastasis^33^. Based on these findings, which suggested the oncogenic role of HDAC7, our study in non-small cell lung cancer (NSCLC) confirmed that HDAC7 deacetylated β-catenin and facilitated its nuclear import and activated FGF18 expression by binding to TCF4^34^. It has been reported that HDAC7 promotes lung tumourigenesis by inhibiting Stat3 activation via its deacetylation Stat3^18^. In the present study, we found that overexpression of HDAC7 significantly enhanced the proliferation and migration abilities of LUAD cells *in vivo* and *in vitro*. The relationship between DNMT3a and HDAC7 has not been reported thus far. The present study found that overexpression of HDAC7 was closely associated with poor prognosis in LUAD patients and that DNMT3a/HDAC7 cooverexpression was dramatically correlated with the poorest prognosis in a subset of LUAD patients. The positive correlation of DNMT3a expression with HDAC7 expression in LUAD was also supported by the results of gene expression profiling interactive analysis of TCGA data. Therefore, we explored whether DNMT3a regulates HDAC7 in LUAD cells and found that DNMT3a clearly upregulated HDAC7.

EMT is the key biological event in tumour cell invasion and metastasis. Epithelium-derived tumour cells undergoing EMT lose cell-to-cell contact and polarity, acquiring migration and invasion capability^32^. Studies have shown that DNMT3a is an essential factor for induction of EMT in prostate cancer, as it ensures the *de novo* methylation required for EMT^35^, ^36^. This DNMT3a-dependent methylation silences epithelial maintenance genes and EMT-counteracting genes, such as E-cadherin and GRHL2, which are direct repressors of ZEB1, the key transcription factor for EMT and stemness^35^. Mancini *et al.* demonstrated the ability of miR-429 to target the DNMT3a 3'UTR and modulate the expression of ZEB1 in prostate cancer^36^. In the present study, we observed that DNMT3a overexpression significantly increased but DNMT3 knockdown decreased the migration and invasion of LUAD cells. Then, the role of DNMT3a in promoting tumour metastasis was confirmed in a nude mouse lung metastasis model. RNA-seq-based transcriptome analysis of DNMT3a knockdown LUAD cell lines combined with GO functional enrichment analysis suggested that decreased ZEB1 mRNA expression was positively correlated with DNMT3a knockdown. Subsequent qRT‒PCR and western blot analyses confirmed, at mRNA and protein expression levels, a similar trend in ZEB1 in the DNMT3a knockdown LUAD cells, and the opposite trend was observed in the DNMT3a overexpression LUAD cells. Moreover, gene expression profiling interactive analysis with data from TCGA datasets demonstrated that DNMT3a expression was positively correlated with HDAC7 expression and that HDAC7 expression was positively correlated with ZEB1 expression in LUAD. Collectively, these results indicated that DNMT3a overexpression may regulate EMT and metastasis through ZEB1 and suggested that the mechanism by which DNMT3a regulates ZEB1 is worth further exploration. The results of IHC staining analysis of xenografts from nude mice in shDNMT3a group and corresponding control group also confirmed that DNMT3a was positively correlated with HDAC7, and HDAC7 was positively correlated with ZEB1 *in vivo*. Similarly, downregulation of E-cadherin expression by DNMT3a-HDAC7 axis was also demonstrated. This result also reinforces the argument that this signaling pathway is likely to promote tumour metastasis by affecting EMT.

Based on the above results, we hypothesized that DNMT3a overexpression can activate ZEB1 through upregulating HDAC7 to enhance the metastasizing ability of LUAD cells. To verify this hypothesis, we established LUAD cell lines with HDAC7 knockdown and overexpression by lentiviral transduction, subsequent western blot analyses demonstrated that ZEB1 protein levels were significantly decreased in HDAC7 knockdown LUAD cells, and markedly elevated in HDAC7 overexpression LUAD cells. Furthermore, we upregulated HDAC7 in DNMT3a knockdown LUAD cells and downregulated HDAC7 in DNMT3a overexpression LUAD cells by lentivirus infection. Subsequent western blot showed that HDAC7 overexpression or knockdown could partially reverse the DNMT3a-induced ZEB1 proteins changes. Furthermore, we tried to knockdown ZEB1 in LUAD cells and found that it had no impact on HDAC7 expression.

As a downstream gene of various growth factor receptors, proto-oncogene c-Myc usually functions as a coding transcription factor to promote cellular responses in response to the interaction between growth factors and their receptors^37^. The proto-oncogene c-Myc is considered a critical transcription factor for cancer cells to acquire the long-term proliferative capacity, since it helps accelerate G1-S cell cycle transition through both transcriptional activation and repression^38^. Inactivation and inhibition of c-Myc can induce tumour regression by restoring the normal cell checkpoint mechanism or inducing proliferation arrest, cell senescence and apoptosis^39^. Studies have found that c-Myc is overexpressed in 50%-75% of non-small cell lung cancer, and multiple activation pathways of c-Myc overexpression have been confirmed^37^. In our previous study, we found that c-Myc was upregulated via overexpression of HDAC7, which facilitated the escape of tumour cells from senescence and promotes their growth^17^. Consistently, Zhu *et al.* found HDAC7 silencing blocked cell cycle progression via inhibiting c-Myc expression in the Hela and MCF-7 cells, revealing that oncogenic actions of HDAC7 are dependent on the c-Myc amplification^40^. Previous studies suggested that amplification of Myc, which has 3 paralogues, c-Myc, n-Myc and l-Myc, is present in numerous solid tumours and that Myc overexpression is significantly correlated with poor prognosis via DNMT3a overexpression^41^. Therefore, we further hypothesized that DNMT3a overexpression can activate c-Myc through upregulating HDAC7 to enhance the proliferation ability of LUAD cells. And the results of both *in vitro* and *in vivo* experiments confirm this hypothesis. A significant positive correlation between c-Myc and DNMT3a in protein expression were validated by western blotting. Likewise, the results of IHC staining analysis of xenografts from nude mice in shDNMT3a group and corresponding control group also confirmed this similar positive correlation trend between c-Myc and DNMT3a *in vivo*. Consistently, we regulated reversely HDAC7 in DNMT3a overexpression or knockdown LUAD cells by lentivirus infection, HDAC7 overexpression or knockdown could partially reverse the DNMT3a-induced c-Myc proteins changes, which was confirmed by western blotting. Similar to c-Myc, CDK6 is also a key molecule regulating cell entry into S phase. In this study, we found that DNMT3a-HDAC7 axis also regulates CDK6 expression. This also suggests that the DNMT3a-HDAC7 signaling axis may promote tumour proliferation mainly by regulating the tumour cell cycle, which also needs to be further confirmed.

Given the potential plasticity of epigenetic regulation and its link to tumour occurrence and progression, epigenetic therapy is among the most promising targeted therapies in terms of both treatment and reversibility of drug resistance. Epigenetic drugs act on the enzymes necessary for the establishment and maintenance of epigenetic modifications, reactivating epigenetically silenced tumour suppressor genes (TSGs) and DNA repair genes, with the main therapeutic strategy being the inhibition of DNMTs and HDACs^42^. DNMT inhibitors (DNMTi) block DNA methylation by replacing cytosines during DNA replication and covalently bind to DNMTs to inhibit their activity and restore the function of aberrantly silenced genes. At the chromatin level, histone acetylation is catalysed by histone acetyltransferases and HDACs^43^. Such modifications occur on amino acid residues (such as lysine) at histone tails. HDAC inhibitors (HDACi) increase histone acetylation in cells, thus increasing the expression levels of TSGs, such as p21, to inhibit the proliferation of tumour cells^44^. Accumulating evidence suggests that HDACs are involved in the initiation and/or maintenance of the inhibition of genes with DNA hypermethylation^45^ and that interactions between DNA methylation and histone deacetylation enhance TSG silencing, further suggesting that dysregulation of either modification may be a major driver of tumourigeneses and tumour survival. This phenomenon also presents a challenge to the durability of DNMTi and HDACi when used as single agents. Studies have shown that simultaneous targeting of DNA methylation and histone deacetylation leads to additive or synergistic effects to reactivate abnormally silenced genes^46^. Moreover, treatment with an HDAC inhibitor after a DNA demethylating agent has been reported to result in synergistic benefits on gene re-expression *in vitro* as well as improved antitumour effects in clinical trials^47^. A versatile DNMT and HDAC inhibitor, C02S, was developed to treat breast cancer and has been shown to reverse aberrant methylation and acetylation at the cellular level and to increase the expression of tumour suppressor proteins, ultimately suppressing tumour cell proliferation *in vitro* and tumour growth *in vivo*^48^. Our results suggested that DNMT3a regulated HDAC7 and formed a positive feedback loop with it, thus promoting the malignant progression of LUAD *in vivo* and *in vitro*. This regulatory mechanism also suggests the potential for the development of agents simultaneously targeting DNMT and HDAC to improve the efficacy of anticancer epigenetic therapy.

Despite these interesting findings in this study, some limitations should be noted. First, as the key question of our current research centres on the role and mechanism of DNMT3a, the specific mechanism of DNMT3a methyltransferase activity in this signaling pathway, such as whether DNMT3a directly acts on some TSG promoter regions to hypermethylate them, thereby regulating the downstream signalling of HDAC7, was not thoroughly investigated. In addition, epigenetic regulatory agents such as DNMTi and HDACi have been reported to alter the expression of genes involved in immune checkpoint pathways and enhance immunosuppression^49^. In the present study, the effects of a specific DNMT inhibitor and HDAC inhibitor on the proliferation and migration of LUAD cells were studied. However, the effects of these two inhibitors in combination or alone on the expression of genes related to immune checkpoint pathways in LUAD cells remains unclear. Therefore, we aim to answer these important questions in future studies.

## 5. Conclusions

In conclusion, our studies confirmed that DNMT3a acted as a tumour promoter to induce malignant progression of LUAD by upregulating HDAC7 and further inducing the upregulation of ZEB1 and c-Myc. In addition, we demonstrated a positive feedback loop between DNMT3a and HDAC7 and found that overexpression of either gene was associated with poor prognosis in LUAD patients. Therefore, targeting DNMT3a along with HDAC7 might be a promising therapeutic strategy for LUAD.

## Supplementary Material

Supplementary methods and figures.

## Figures and Tables

**Table 1:** Correlation of DNMT3a and HDAC7 expression with clinicopathological characteristics of LUAD patients.

Clinicopathological variables	n	DNMT3a expression		*p* value	HDAC7 expression		*p* value
		High	Low		High	Low	
SEX							
Female	43	18	25	0.160	17	26	0.074
Male	76	42	34		43	33	
Age							
≥ 60	60	31	29	0.648	32	28	0.522
< 60	59	29	30		28	31	
Smoking history							
Never	61	30	31	0.642	29	32	0.519
Ever	58	30	28		31	27	
Maximal tumour size							
< 5 cm	58	20	38	<0.01	23	35	0.054
≥ 5 cm	61	40	21		37	24	
T stage							
T1-T2	65	19	46	<0.01	22	43	<0.01
T3-T4	54	41	13		38	16	
Lymphatic invasion							
N0	45	14	31	<0.01	13	32	<0.01
N1-N3	74	46	28		47	27	
Distant metastasis							
No	116	57	59	0.244	57	59	0.244
Yes	3	3	0		3	0	
TNM stages							
I-II	53	9	44	<0.01	10	43	<0.01
III-IV	66	51	15		50	16	
Tumour differentiation							
High and moderate	85	34	51	<0.01	36	49	<0.01
Poor	34	26	8		24	10	

**Table 2:** Univariate and multivariate analysis of prognostic factors for LUAD patients' survival.

Clinicopathological variables	Univariate analysis			Multivariate analysis		
	HR	95% CI	*p* value	HR	95% CI	*p* value
SEX (Female/Male)	1.006	0.675-1.500	0.975			
Age (≥ 60/< 60 years)	0.739	0.485-1.126	0.160			
Smoking history (Never/Ever)	1.212	0.813-1.806	0.345			
Maximal tumour size (<5/≥5cm)	1.438	0.964-2.145	0.075			
T stage (T1-T2/T3-T4)	2.613	1.738-3.930	<0.01			
Lymphatic invasion (N0/N1-N3)	1.674	1.097-2.555	0.017			
Distant metastasis (No/Yes)	4.372	1.349-14.170	0.014			
TNM stages (I-II/III-IV)	4.581	2.905-7.223	<0.01	2.596	1.265-5.327	<0.01
Tumour differentiation (High and moderate/Poor)	3.988	2.549-6.239	<0.01	2.752	1.722-4.398	<0.01
Tumour DNMT3a expression (Low/High)	7.887	4.724-13.169	<0.01	2.699	1.372-5.311	<0.01
Tumour HDAC7 expression (Low/High)	5.967	3.694-9.639	<0.01	2.247	1.238-4.078	<0.01

## Abbreviations

AML: Acute myeloid leukaemia
CDK4: Cyclin-dependent kinase 4
CDK6: Cyclin-dependent kinase 6
DNMT3a: DNA methyltransferase 3 alpha
EMT: Epithelial-to-mesenchymal transition
ESCC: Esophageal squamous cell carcinoma
HDAC7: Histone deacetylase 7
IHC: Immunohistochemical
IP: Immunoprecipitation
LUAD: Lung adenocarcinoma
MBD: Methyl-CpG binding domain
qRT-PCR: Quantitative real-time polymerase chain reaction
SAM: S-adenosyl methionine
ZEB1: Zinc finger E-box-binding homeobox 1
