# Supplementary material for: DNMT3a promotes LUAD cell proliferation and metastasis by activating the HDAC7 signalling pathway
Source: Int J Biol Sci. 2025 Jan 27;21(4):1585–602. doi: 10.7150/ijbs.96509 (PMC11844290; doi:10.7150/ijbs.96509)
Supplement: Supplementary file 1 — Supplementary methods and figures. [file ijbsv21p1585s1.pdf]

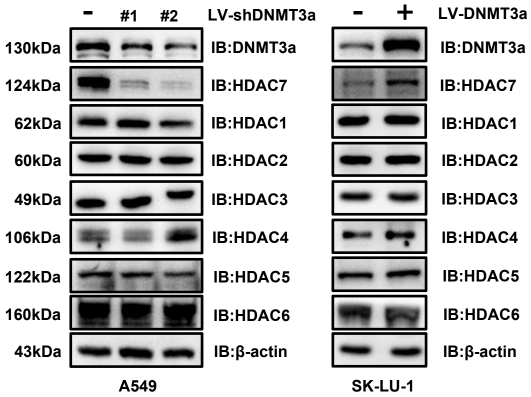
-
#1
#2
LV-shDNMT3a
-
+
LV-DNMT3a
130kDa
IB:DNMT3a
124kDa
IB:HDAC7
62kDa
IB:HDAC1
60kDa
IB:HDAC2
49kDa
IB:HDAC3
106kDa
IB:HDAC4
122kDa
IB:HDAC5
160kDa
IB:HDAC6
43kDa
IB:β-actin
A549
SK-LU-1

Fig. S1 The relationship between DNMT3a and HDACs. Representative results of western blot of HDACs expression in DNMT3a knockdown and overexpression LUAD cells.

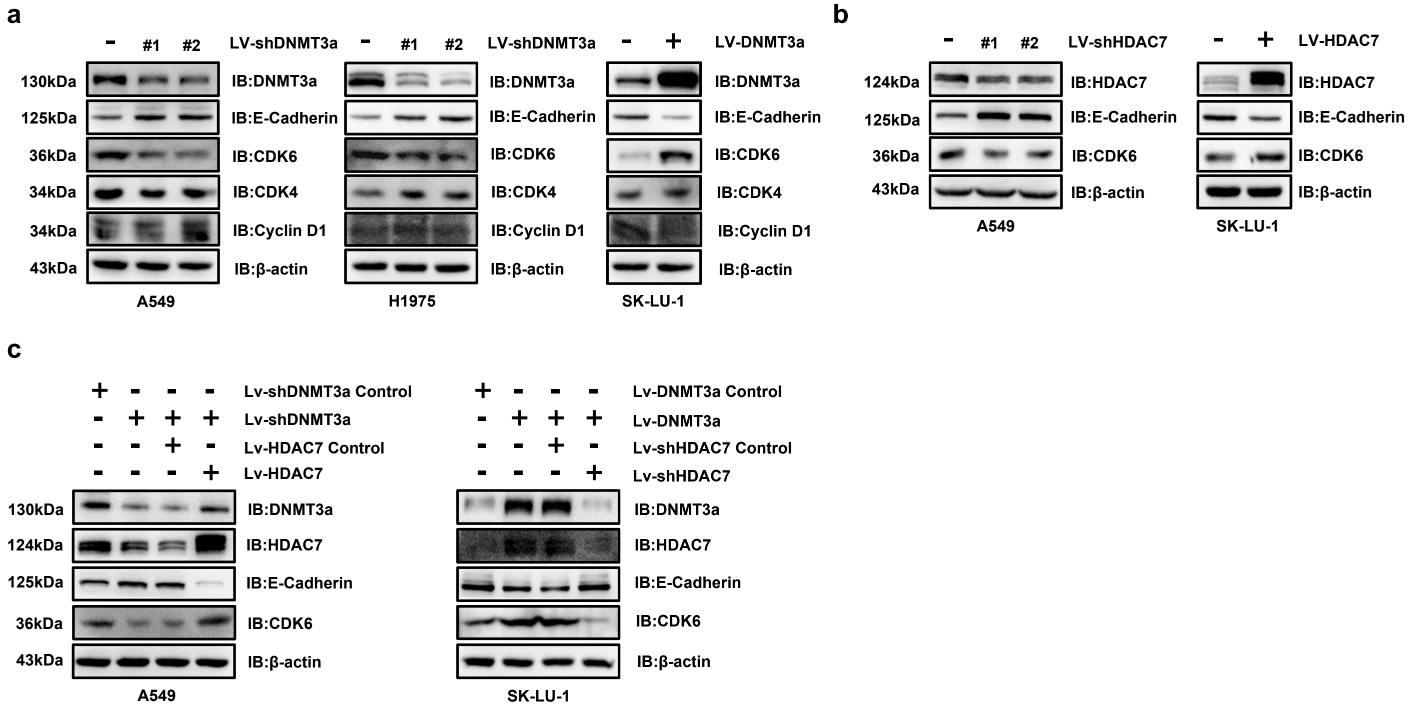
a
- #1 #2 LV-shDNMT3a
- #1 #2 LV-shDNMT3a
- + LV-DNMT3a
130kDa IB:DNMT3a
125kDa IB:E-Cadherin
36kDa IB:CDK6
34kDa IB:CDK4
34kDa IB:Cyclin D1
43kDa IB:β-actin
A549
H1975
SK-LU-1
b
- #1 #2 LV-shHDAC7
- + LV-HDAC7
124kDa IB:HDAC7
125kDa IB:E-Cadherin
36kDa IB:CDK6
43kDa IB:β-actin
A549
SK-LU-1
c
Lv-shDNMT3a Control
Lv-shDNMT3a
Lv-HDAC7 Control
Lv-HDAC7
Lv-DNMT3a Control
Lv-DNMT3a
Lv-shHDAC7 Control
Lv-shHDAC7
130kDa IB:DNMT3a
124kDa IB:HDAC7
125kDa IB:E-Cadherin
36kDa IB:CDK6
43kDa IB:β-actin
A549
SK-LU-1

Fig. S2 DNMT3a or HDAC7 knockdown upregulates the expression of E-cadherin and CDK6 in LUAD cells. a Representative results of western blot of E-cadherin, CDK6, CDK4 and cyclin D1 expression in DNMT3a knockdown and overexpression LUAD cells. **b** Representative results of western blot of E-cadherin and CDK6 expression in HDAC7 knockdown and overexpression LUAD cells. **c** Representative western blot analysis of DNMT3a, HDAC7, E-cadherin and CDK6 expression in the indicated groups. β-actin was used as an internal control.

a

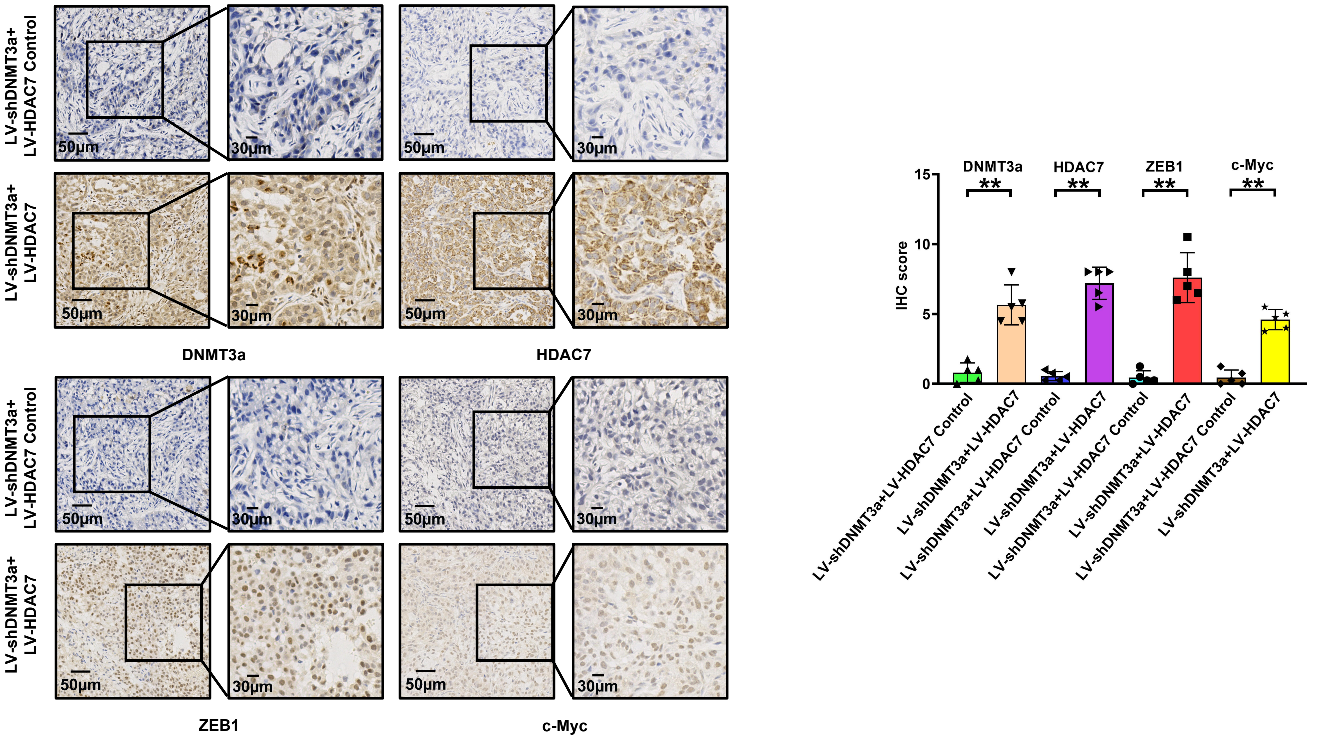
LV-shDNMT3a+
LV-HDAC7 Control
LV-shDNMT3a+
LV-HDAC7
50μm
30μm
DNMT3a
HDAC7
ZEB1
c-Myc
IHC score
DNMT3a
HDAC7
ZEB1
c-Myc
**
LV-shDNMT3a+LV-HDAC7 Control
LV-shDNMT3a+LV-HDAC7

b

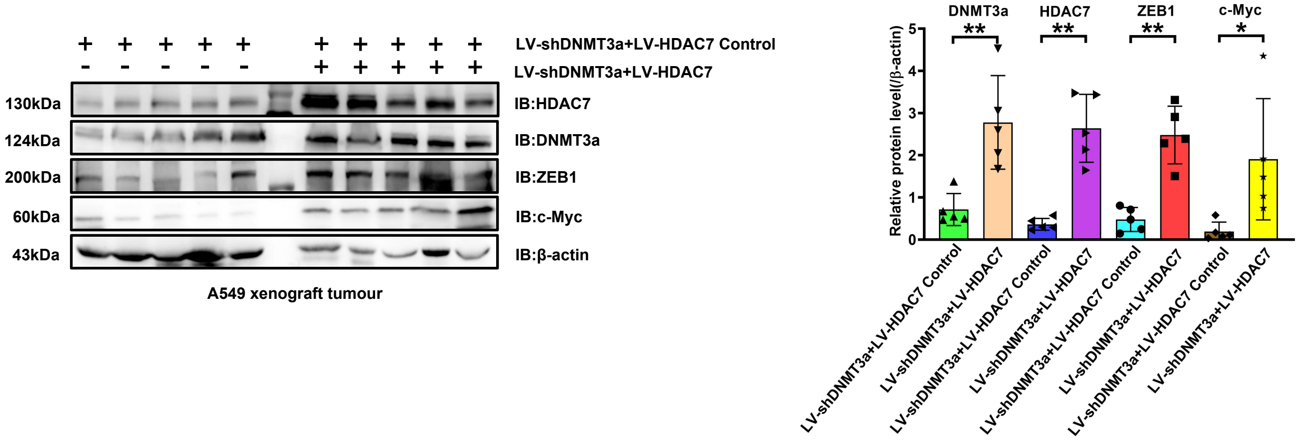
LV-shDNMT3a+LV-HDAC7 Control
LV-shDNMT3a+LV-HDAC7
130kDa
124kDa
200kDa
60kDa
43kDa
IB:HDAC7
IB:DNMT3a
IB:ZEB1
IB:c-Myc
IB:β-actin
A549 xenograft tumour
Relative protein level(/β-actin)
DNMT3a
HDAC7
ZEB1
c-Myc
**
*
LV-shDNMT3a+LV-HDAC7 Control
LV-shDNMT3a+LV-HDAC7

Fig. S3 HDAC7 overexpression partially reversed the effects of DNMT3a knockdown. **a** Representative images and statistical analysis of DNMT3a, HDAC7, ZEB1 and c-Myc IHC staining in xenograft tumours from nude mice in the indicated group. Scale bars, 50 μm and 30 μm (inset). **b** Representative results of western blot analysis of DNMT3a, HDAC7, ZEB1 and c-Myc expression in xenograft tumour cells from nude mice in the indicated group. β-actin was used as an internal control. * $p < 0.05$, ** $p < 0.01$.

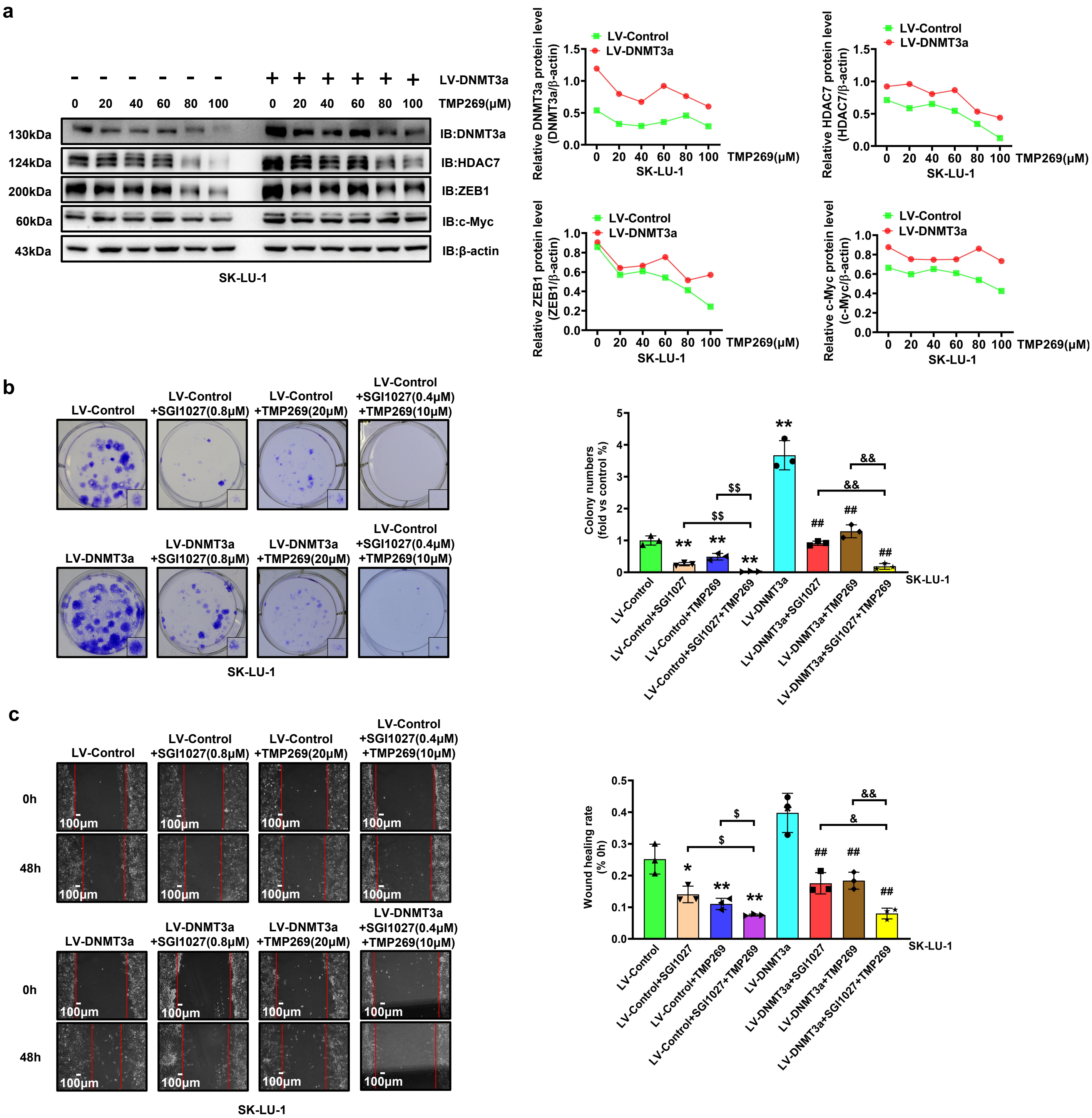
a
LV-DNMT3a
TMP269(μM)
0 20 40 60 80 100
130kDa
124kDa
200kDa
60kDa
43kDa
IB:DNMT3a
IB:HDAC7
IB:ZEB1
IB:c-Myc
IB:β-actin
SK-LU-1
LV-Control
LV-DNMT3a
Relative DNMT3a protein level (DNMT3a/β-actin)
Relative HDAC7 protein level (HDAC7/β-actin)
Relative ZEB1 protein level (ZEB1/β-actin)
Relative c-Myc protein level (c-Myc/β-actin)
b
LV-Control
LV-Control +SGI1027(0.8μM)
LV-Control +TMP269(20μM)
LV-Control +SGI1027(0.4μM) +TMP269(10μM)
LV-DNMT3a
LV-DNMT3a +SGI1027(0.8μM)
LV-DNMT3a +TMP269(20μM)
LV-DNMT3a +SGI1027(0.4μM) +TMP269(10μM)
SK-LU-1
Colony numbers (fold vs control %)
LV-Control+SGI1027
LV-Control+TMP269
LV-Control+SGI1027+TMP269
LV-DNMT3a+SGI1027
LV-DNMT3a+TMP269
LV-DNMT3a+SGI1027+TMP269
c
0h
48h
100μm
Wound healing rate (% 0h)

Fig. S4 TMP269 reversed the DNMT3a-induced changes in the expression of proteins in LUAD cells. a Representative western blot and statistical analysis of DNMT3a, HDAC7, ZEB1 and c-Myc expression in DNMT3a overexpression LUAD cells and control cells after treatment with TMP269 for 48 h. β-actin was used as an internal control. **b** Representative images and statistical analysis of the colony formation assay in the indicated groups after treatment with SGI1027 and/or TMP269 for 48 h. Colonies were visualized by crystal violet staining. **c** Representative wound healing assay images and statistical analysis in the indicated groups after treatment with SGI1027 and/or TMP269 for 48 h. The migration ability was quantified as the mean scratch area at each time point. The initial scratch area (0 h) was set as 100%. Scale bars, 100 μm (inset). * $p < 0.05$ vs. the LV-Control group, ** $p < 0.01$ vs. the LV-Control group, $^{\#\#}$ $p < 0.01$ vs. the LV-DNMT3a group, $^{\$\$}$ $p < 0.01$ vs. the LV-Control+SGI1027+TMP269 group, $^{\&\&}$ $p < 0.01$ vs. the LV-DNMT3a+SGI1027+TMP269 group.

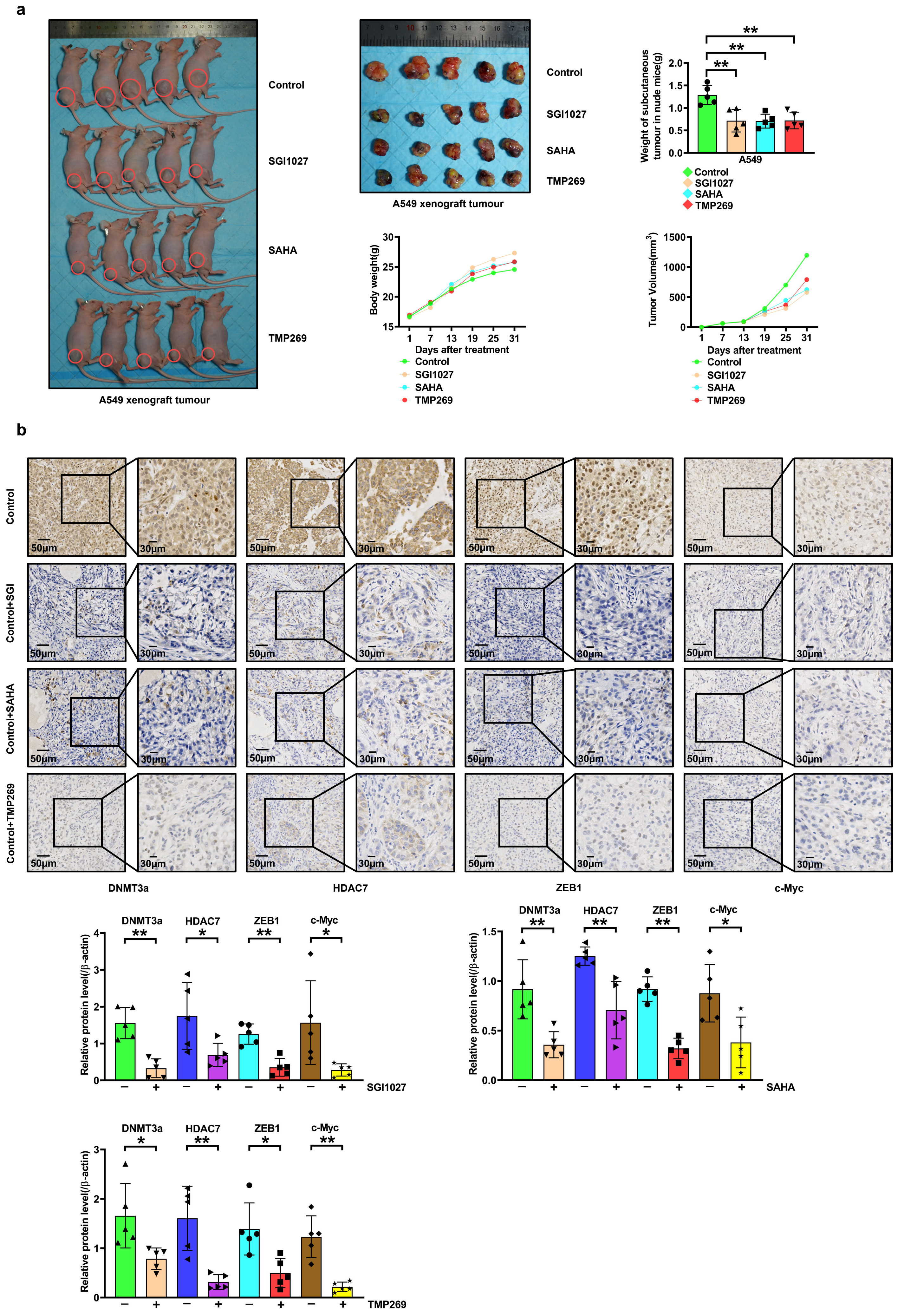
a
Control
SGI1027
SAHA
TMP269
A549 xenograft tumour
Control
SGI1027
SAHA
TMP269
A549 xenograft tumour
Weight of subcutaneous tumour in nude mice(g)
A549
Control
SGI1027
SAHA
TMP269
Body weight(g)
Days after treatment
Control
SGI1027
SAHA
TMP269
Tumor Volume(mm3)
Days after treatment
Control
SGI1027
SAHA
TMP269
b
Control
Control+SGI
Control+SAHA
Control+TMP269
50μm
30μm
DNMT3a
HDAC7
ZEB1
c-Myc
Relative protein level(/β-actin)
DNMT3a
HDAC7
ZEB1
c-Myc
SGI1027
Relative protein level(/β-actin)
DNMT3a
HDAC7
ZEB1
c-Myc
SAHA
Relative protein level(/β-actin)
DNMT3a
HDAC7
ZEB1
c-Myc
TMP269

Fig. S5 DNMT3a and HDAC7 inhibitors decreased LUAD cell progression in vivo. a Gross photograph of subcutaneous xenograft tumours and data showing the changes in subcutaneous tumour weight, tumor volume and nude mouse body weight in each group. **b** Representative images and statistical analysis of DNMT3a, HDAC7, ZEB1 and c-Myc IHC staining in xenograft tumours from nude mice in the indicated group. Scale bars, 50 μm and 30 μm (inset). * $p < 0.05$, ** $p < 0.01$.

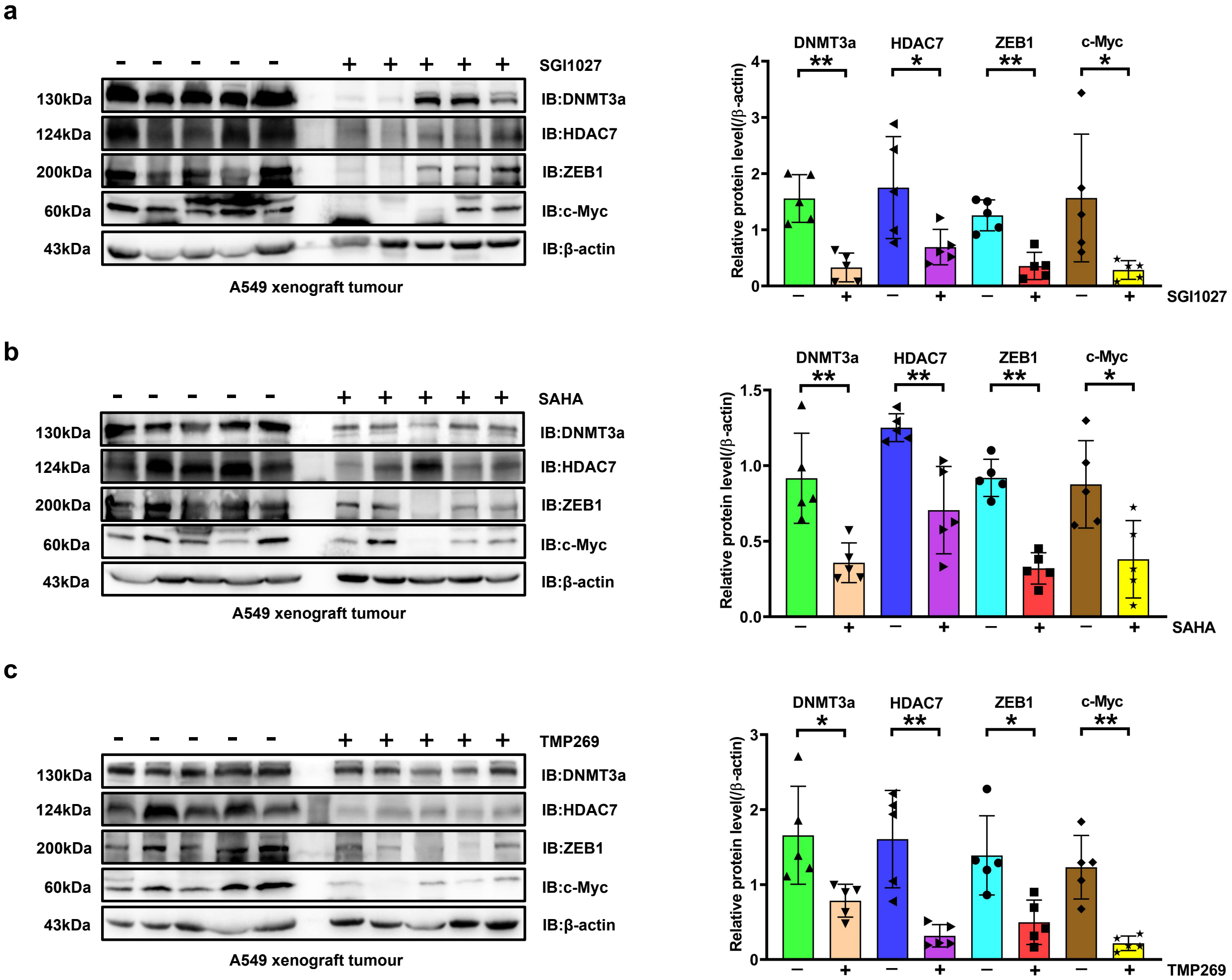
a
- - - - - + + + + + SGI1027
130kDa IB:DNMT3a
124kDa IB:HDAC7
200kDa IB:ZEB1
60kDa IB:c-Myc
43kDa IB:β-actin
A549 xenograft tumour
DNMT3a
HDAC7
ZEB1
c-Myc
**
*
**
*
Relative protein level(/β-actin)
0
1
2
3
4
− + − + − + − + SGI1027
b
- - - - - + + + + + SAHA
130kDa IB:DNMT3a
124kDa IB:HDAC7
200kDa IB:ZEB1
60kDa IB:c-Myc
43kDa IB:β-actin
A549 xenograft tumour
DNMT3a
HDAC7
ZEB1
c-Myc
**
**
**
*
Relative protein level(/β-actin)
0.0
0.5
1.0
1.5
− + − + − + − + SAHA
c
- - - - - + + + + + TMP269
130kDa IB:DNMT3a
124kDa IB:HDAC7
200kDa IB:ZEB1
60kDa IB:c-Myc
43kDa IB:β-actin
A549 xenograft tumour
DNMT3a
HDAC7
ZEB1
c-Myc
*
**
*
**
Relative protein level(/β-actin)
0
1
2
3
− + − + − + − + TMP269

Fig. S6 SGI1027, SAHA and TMP269 reversed the DNMT3a-induced changes in the expression of proteins in vivo. a Representative western blot and statistical analysis of DNMT3a, HDAC7, ZEB1 and c-Myc expression in xenograft tumours from nude mice after treatment with SGI1027. **b** Representative western blot and statistical analysis of DNMT3a, HDAC7, ZEB1 and c-Myc expression in xenograft tumours from nude mice after treatment with SAHA. **c** Representative western blot and statistical analysis of DNMT3a, HDAC7, ZEB1 and c-Myc expression in in xenograft tumours from nude mice after treatment with TMP269. β-actin was used as an internal control. * $p < 0.05$, ** $p < 0.01$.

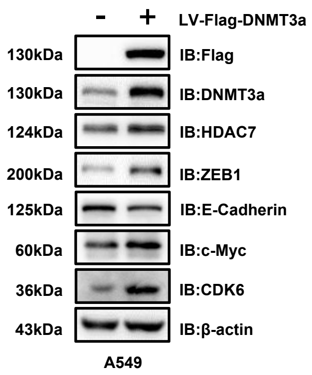
-
+
LV-Flag-DNMT3a
130kDa
IB:Flag
130kDa
IB:DNMT3a
124kDa
IB:HDAC7
200kDa
IB:ZEB1
125kDa
IB:E-Cadherin
60kDa
IB:c-Myc
36kDa
IB:CDK6
43kDa
IB:β-actin
A549

Fig. S7 Overexpression of DNMT3a upregulates the expression of HDAC7, ZEB1 and c-Myc in LUAD. Representative results of western blot of HDAC7, DNMT3a, ZEB1, and c-Myc expression in DNMT3a overexpression LUAD cells. β-actin was used as an internal control.

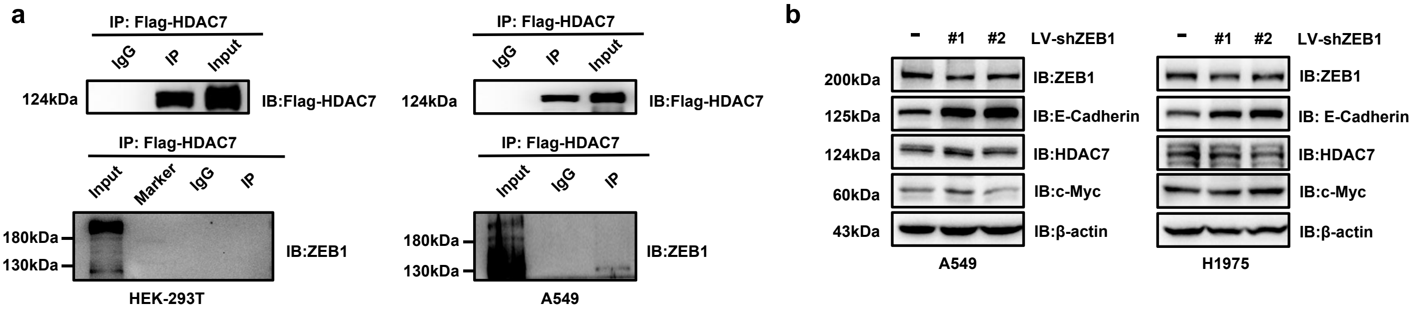
a
IP: Flag-HDAC7
IgG
IP
Input
124kDa
IB:Flag-HDAC7
IP: Flag-HDAC7
IgG
IP
Input
124kDa
IB:Flag-HDAC7
IP: Flag-HDAC7
Input
Marker
IgG
IP
180kDa
130kDa
IB:ZEB1
HEK-293T
IP: Flag-HDAC7
Input
IgG
IP
180kDa
130kDa
IB:ZEB1
A549
b
-
#1
#2
LV-shZEB1
200kDa
IB:ZEB1
125kDa
IB:E-Cadherin
124kDa
IB:HDAC7
60kDa
IB:c-Myc
43kDa
IB:β-actin
A549
-
#1
#2
LV-shZEB1
IB:ZEB1
IB: E-Cadherin
IB:HDAC7
IB:c-Myc
IB:β-actin
H1975

Fig. S8 HDAC7 and ZEB1 do not interact directly, and ZEB1 does not regulate the expression of HDAC7 in LUAD. a Co-IP analysis and western blotting were performed to confirm that there was no interaction between HDAC7 and ZEB1 in either HEK-293T or A549 cells. **b** Representative western blot analysis of ZEB1, E-cadherin, HDAC7, and c-Myc expression in ZEB1 knockdown LUAD cells. β-actin was used as an internal control.

Supplementary Information

Screening criteria for clinical patients

The patients with LUAD who underwent surgical treatment at Department of Thoracic Surgery, Tangdu Hospital, Air Force Military Medical University from May 2009 to January 2014 were enrolled in this retrospective study.Patients were screened according to the following criteria: (1) Pathologically confirmed LUAD without special pathological components such as adenosquamous carcinoma; (2) None of the patients had received chemotherapy, radiotherapy, targeted therapy, immunotherapy and other tumor-related therapies before surgery. (3) The patient had no tumor in other organs. All patients had signed the informed consent, and the study had been approved by the Ethics Committee of the Second Affiliated Hospital of Air Force Medical University.

Follow-up content and requirements of clinical patients

The follow-up contents and requirements were as follows: (1) Basic information of patients, including age, gender, smoking history and other basic information; (2) Surgical and pathological information: including operation time, postoperative pathological results, pathological grade, TNM clinical stage of lung cancer according to the 8th AJCC edition (including T stage, N stage, M stage), etc. (3) Follow-up content: including overall survival, medication and physical examination; (4) Follow-up methods: outpatient and telephone follow-up; (5) Follow-up cycle: once every 3 months within 2 years after surgery, once every 6 months after 2 years; (6) Follow-up time: up

to January 2019 or death of the patient, whichever came first. The study was conducted in accordance with the Declaration of Helsinki and clinical guidelines.
